# Notch1 activation of Jagged1 contributes to differentiation of mesenchymal stem cells into endothelial cells under cigarette smoke extract exposure

**DOI:** 10.1186/s12890-022-01913-3

**Published:** 2022-04-11

**Authors:** Yi Cheng, Wen Gu, Guorui Zhang, Xuejun Guo

**Affiliations:** 1 Department of Respiratory Medicine, Xinhua Hospital Affiliated to Shanghai Jiao Tong University School of Medicine, 1665 KongJiang Road, Shanghai, 200092 China; 2 Department of Respiratory Medicine, The First Affiliated Hospital of Zhengzhou University, Zhengzhou, China

**Keywords:** Differentiation, Mesenchymal stem cells, Jagged1, Notch1, Endothelial cell

## Abstract

**Background:**

Mesenchymal stem cells (MSCs) have shown therapeutic potential for engraftment to, differentiation into, endothelial cells (ECs). However, low-efficiency yields hinder their use as ECs for therapeutic vascularization.

**Methods:**

The Notch1 signaling pathway is key to optimal pulmonary development. Recent evidence has shown that this pathway participated in angiogenesis. Herein, we found that in MSCs, Jagged1 was a target for Notch 1, resulting in a positive feedback loop that propagated a wave of ECs differentiation.

**Results:**

In vitro, Jagged1 was found to be activated by Notch1 in MSCs, resulting in the RBP-Jκ-dependent expression of Jagged1 mRNA, a response that was blocked by Notch1 inhibition. Notch1 promoted the formation of cord-like structures on Matrigel. However, cigarette smoke extract inhibited this process, compared to that in control groups. Moreover, Notch1-overexpressing cells upregulated the expressing of *HIF-1α* gene. The HIF-1α was an angiogenic factor that clustered with Notch1, underscoring the critical role of Notch1 pathway in vessel assembly. Interestingly, this was abrogated by incubation with Notch1 shRNA.

**Conclusions:**

Notch signaling pathway promotes differentiation of MSCs in to ECs. It also regulates angiogenesis and transcription of specific markers on ECs. These results provide a mechanism that regulates differentiation of MSCs into ECs phenotypes.

**Supplementary Information:**

The online version contains supplementary material available at 10.1186/s12890-022-01913-3.

## Introduction

Chronic obstructive pulmonary disease (COPD) is a complex ailment involving loss of lung microvessel. Cigarette smoke (CS) is considered the chief causative agent in the development of COPD. Cigarette smoke has shown direct effects on the pulmonary vasculature by increased cell senescence and altered vascular tone which contributes to pulmonary arterial remodeling [1]. Excessive endothelial apoptosis was identified as the principal feature that might contribute to emphysema development [2]. Although the hypothesis of protease–antiprotease imbalance is widely accepted, endothelial cells (ECs) apoptosis is the most important factor contributing to alveolar damage, leading to emphysema. In a preclinical study, endothelial apoptosis resulted in enlarged air spaces in rats [3]. Supporting this, increase in apoptosis of ECs has also been observed in the lungs of COPD patients [4]. Pulmonary microvascular blood flow (PMBF) is low in COPD, even in regions of the lung without overt emphysema. This suggests PMBF participates in the pathology of small airway disease [5]. Thus, the hypothesis that blood vessel paucity participates in lung parenchymal loss in emphysema was proposed. Mesenchymal stem cells (MSCs) are reliable targets as ECs sources and have been shown to promote vascularity during coronary diseases [6]. Previous studies demonstrated that although MSCs-derived ECs can be generated from basic fibroblast growth factor (bFGF), vascular endothelial growth factor (VEGF) in vivo and several cytokines [7, 8], the efficiency of this process is sub-optimal for advanced therapies. The results of Wang et al. found that promoting the differentiation of MSCs into endothelial cells efficiently improved the possibility of application in the repair of peripheral arterial disease [9]. Numerous signaling pathways such as VEGF and transforming growth factor, FGF, angiopoietin/Tie signaling, the ephrin/Eph receptor, among others, have been proposed to regulate differentiation of MSCs into ECs. Of those, Notch signaling regulates cellular differentiation in multiple bilaterian tissue types [10–12]**.** Interaction of Notch ligand–receptor results in proteolytic cleavage and plasma membrane produce Notch intracellular domain (NICD). In the canonical Notch pathway, Notch signaling regulates the expression of downstream genes via the RBP-Jκ transcription factor [13]. The nuclear NICD regulates expression of downstream targets [14]. Homozygous knockouts of several Notch pathway components result in embryonic lethality with vascular remodeling defects [15]. Furthermore, angiogenesis is disrupted in these mutants, underlining the critical role Notch signaling plays in angiogenesis. Unfortunately, how it participates in MSCs differentiation into ECs remains unclear. Herein, a possible role for Notch in modulating MSCs differentiation into ECs was examined. To ascertain the particular biological effects of CS on MSCs, we used cigarette smoke extract (CSE) to mimic the CS microenvironment in vitro*.* We hypothesized that the function of MSCs differentiation into ECs would be improved by Notch1 signaling activation under CSE exposure. To test this hypothesis, we investigated the role of Notch1 on MSCs differentiation into ECs in the CS microenvironment. Furthermore, the role of Notch in regulation of this process was confirmed dependent on RBP-Jκ. These results shed light on molecular mechanisms of Notch1 to promote ECs differentiation for stem cell-based and provided potential therapies for COPD.

## Materials and methods

### Reagents and antibodies

Fetal bovine serum (FBS), Dulbecco’s modified Eagle’s medium: Nutrient mixture F-12 (D-MEM/F-12, Gibco, USA), bovine serum albumin (BSA), and trypsin/EDTA (Gibco-BRL); EGM-2 Bullet Kit (mixture of ascorbic acid, hydrocortisone, EGF, IGF-I, heparin, VEGF, and FGF2,; Lonza); insulin-transferrin-selenium sodium pyruvate (ITS; Invitrogen); Jagged1 Fc Chimera (599-JG; R&D Systems); rat immunoglobulin G (IgG) (I4131; Sigma Aldrich); PE-mouse anti-Rat CD31 (BD Pharmingen™); anti-Hif-1α antibody (Proteintech); R-PE-conjugated Donkey Anti-Goat IgG(H + L) (Proteintech); anti-Jagged-1 antibody (Affinity); anti-N1ICD antibody (CST); anti-Hey1 antibody (Abcam); anti-VEGF receptor2 antibody (Abcam); anti-Von Willebrand factor (vWF) antibody (Abcam); goat anti-rabbit IgG Alexa Fluor 594 (Invitrogen); TRIzol (Takara, Biotechnology, Dalian, China); 4,6-diamidino-2-phenylindole (DAPI; Molecular Probes); reverse Q-PCR kit, and RT-PCR kit, protein lysis buffer kit (Beyotime Biotechnology); anti-actin antibody (Sigma); horseradish peroxidase (HRP)-conjugated goat anti-rabbit and goat anti-mouse IgG (Beyotime Biotechnology); Matrigel (Corning); Dual-GLO Luciferase Assay System (Promega); Fugen6 (Roche).

### Bone marrow MSCs isolation, expansion, and identification

Bone marrow MSCs (BM-MSCs) were isolated as previously described [16]. BM-MSCs were isolated using 4-week-old male Sprague–Dawley rats (Chinese Academy of Science, Shanghai, China). Briefly, 50 mg/kg of sodium pentobarbital was first administered intraperitoneally for anesthetization. (Thereafter, both the femurs and tibia were excised before inserting an 18-gauge needle with a 5.0-mL syringe in to the epiphysis. The rats were sacrificed after MSCs extraction in this study by euthanasia (pentobarbital 100 mg/kg intraperitoneally). The aspirated bone marrow was then re-suspended in DMEM-F12 supplemented with 10% FBS at 2 × 10^7^ cells/mL. The culture was kept at 37 °C in a humidified incubators under 5% carbon (iv) dioxide. After 24 h. Hematopoietic cells that had not adhered to the well surface were washed off using the medium. During expansion, the medium was changed every 3 days. Upon reaching 80–90% confluent, the MSCs became were detached from the well surface using 0.25% trypsin/0.02% EDTA and thereafter re-incubated at a 1:2 dilution. A homogeneous population of MSCs was generated after three passages. To verify the multipotency of cultured MSCs, the cells were cultured in differentiation medium to assess if they had the capacity to differentiate into osteoblasts; adipocytes, moreover, we assessed positive (CD29, CD90, CD73, and CD105) and negative (CD34, CD45, CD80, and CD86) BM-MSCs surface markers (Additional file 2: Fig. S1). All methods were carried out in accordance with the Declaration of Helsinki and the guidelines of the Ethics Committee of Xinhua Hospital affiliated to Shanghai Jiao Tong University School of Medicine, Shanghai, China.

### N1ICD overexpression, Notch1 shRNA lentivirus, and transduction

A lentiviral vector (GV365, Ubi-MCS-3FLAG-CMV-EGFP) containing rat N1ICD-encoding cDNA was produced by GENECHEM (Shanghai, China) for N1ICD overexpression in MSCs (Lenti-N1ICD-MSCs). Rat Notch-1 cDNA (NM_001105721) was generated by PCR using the primer sequences as follows; P1: 5′-GAGGATCCCCGGGTACCGGTCGCCACCATGCGCAAGCGCAGGCGGCAGCATG-3′ and P2: 5′-TCCTTGTAGTCCATACCCTTAAATGCCTCTGGAATGTGGGTG-3′ (Table 1). The melting temperature of the primer sequence was 62℃. Small hairpin RNAs (shRNAs) specifically targeting rat Notch-1 cDNA sequence (NM_001105721) in GenBank were bought from GENECHEM (Shanghai, China). shRNA sequences were biosynthesized and were inserted into the AgeI and EcoRI site of linearized GV248 (hU6-MCS-Ubiquitin-EGFP-IRES-puromycin, GENECHEM, Shanghai). Empty lentivirus vectors GV365 and GV248 were transduced into MSCs as controls (Lenti-V). After transduction, MSCs carrying an empty vector, a Notch-overexpression vector (Lenti-N1ICD), or Notch1 shRNA (Lenti-Notch1-shRNA) were cultured in a mixture of DMEM/F12 in 1:1 and supplemented with 10% FBS and a mixture of 1% streptomycin and penicillin. The cells were kept in incubators with 5% CO_2_ at 37 °C. The cells were used for experiments 72 h post-transfection.

**Table 1:** Primer sequences of rat N1ICD-encoding cDNA

Gene	Primer sequence
Notch1-P1	5′GAGGATCCCCGGGTACCGGTCGCCACCATGCGCAAGCGCAGGCGGCAGCATG3′
Notch1-P2	5′TCCTTGTAGTCCATACCCTTAAATGCCTCTGGAATGTGGGTG3′

### RNA interference of RBP-Jκ

shRNAs specifically targeting rat RBP-J (NM_001106631) or non-targeting controls were bought from GENECHEM (Shanghai, China). shRNA sequences were biosynthesized and were inserted into the AgeI and EcoRI site of linearized GV248(hU6-MCS-Ubiquitin-EGFP-IRES-puromycin, GENECHEM, Shanghai). Before transfection, MSCs were seeded in 12-well plates at 10^5^/mL and maintained in antibiotic free DMEM/F12. For transfection, upon reaching 50–70% density, cells were cultured in serum-free Opti-MEM I medium (Invitrogen, Life Technologies, Carlsbad, California, USA). The aforementioned medium was replaced with culture medium. The MSCs transfection was done with Lipofectamine 2000 reagent (Invitrogen).

### Flow cytometry

MSCs-derived ECs were identified based on the CD31 surface maker protein and flow cytometry. For controls, isotype-identical antibodies were used. The process was carried out with a BD FACS Calibur flow cytometer and analysis was done with Cell Quest software (Becton Dickinson). CD31-positive cell numbers were recorded. The purity assessment of cells was done by flow cytometry as previously reported by Fathi et al. [17].

### Differentiation of MSCs into endothelial cells

CSE contains all of the compounds inhaled by smokers and is frequently added to cells in culture to monitor specific cellular responses [18]. Here, MSCs (2 × 10^4^ cells/cm^2^) were cultured in induction medium supplemented with EGM-2, 2% ITS and EGM-2 Bullet Kit reagents with VEGF and bFGF concentrations of 10 and 2 ng/mL, respectively. Cultures were carried out for 14 days at 37 °C in an atmosphere of 5% CO_2_ and 95% relative humidity. Next, to delineate the impact of CSE and Notch signaling on endothelial differentiation, Lenti-V, Lenti-N1ICD-MSCs, and Lenti-Notch1-shRNA-MSCs were add to the corresponding differentiation medium with or without 2% CSE for various periods up to 14 days. To validate Jagged1 Fc Chimera selectivity, we also tested the effects of IgG, an isotype control, on differentiation. The vehicle for Jagged1 Fc Chimera was PBS. The medium was replaced with fresh medium three times per week.

### Immunofluorescent staining in vitro

After fixation with 4% paraformaldehyde, cells were permeabilized at room temperature for 10 using 0.25% triton X-100 dissolved in PBS and blocked for 40 min at room temperature (RT) using 5% BSA. Thereafter, the cells were incubated at 4 °C overnight with primary antibodies, washed three times using PBS before re-incubation with secondary goat anti-rabbit IgG Alexa Fluor 594 (Invitrogen) for 1 h; the stain was rinsed off using anti-fade 4–6-diamidino-2-phenylindole (DAPI, 0.1 mg/mL). Next, 100 cells/plate were subjectively examined using a fluorescent microscope (Olympus Life Science, Tokyo, Japan). The data was analyzed using Image-Pro Plus software (Media Cybernetics).

### Real-time PCR analyses

Total RNA in undifferentiated/differentiated MSCs was extracted using TRIzol, reverse transcribed into cDNA and amplified based on qPCR using SYBR Green Super Mix (Takara Biotechnology) and rat-specific primers; GAPDH was used as the internal control, Primer sequences were listed in Table 2. Amplification was done using the ABI Prism 7500 Sequence Detection System (Applied Biosystems, Foster City, CA, USA). The amplification conditions included the following; initial denaturation at 95 °C for 30 s, subsequent 40 cycles of denaturation at 95 °C for 5 s, annealing at 60 °C for 34 s, with a final dissociation. The relative amplification of the transcripts was based on 2^−△△^CT method. The results were normalized to untreated controls.

**Table 2:** Primer sequences used for real-time polymerase chain reaction

Gene	Primer sequence	Product length (bp)
Hey1	Forward:5’GCGCGGACGAGAATGGAAA3’ Reverse:5’TCAGGTGATCCACAGTCATCTG3’	231
HeyL	Forward:5’CAGCCCTT CGCAGATGCAA3’ Reverse:5’CCAATCGTCGCAATTCAGAAAG3’	101
Hes3	Forward:5’ TGTCACTGGAGCAGCTAAGGT3’ Reverse:5’ ATCGGCCTTCTCCAGCTTTC3’	80
Hes5	Forward:5’ CGTCAGCTACCTGAAGCACAG 3’ Reverse:5’ CACCACGAGTAACCCTCGCT3’	90
VE-cadherin	Forward:5’ AGATATCCGTGTGGGCAAGC 3’ Reverse:5’ CTGTACTCGCCCTGCATGAT 3’	102
eNOS	Forward:5’ TGTGACCCTCACCGATACAA3’ Reverse:5’ CACAGCCACGTTAATTTCCA3’	117

### Western blotting

Cells were collected and protein was extracted as previously explained by Fathi et al. [19]. In brief, cells were lysed using lysis buffer (Beyotime Institute of Biotechnology) enriched with 1 mmol/L NaF, 1 mmol/L Na_3_VO_4_ and 1 mmol/L PMSF. The concentrations of proteins were quantified using the BCA protein assay. Proteins were separated using 6% and 10% gradient sodium SDA-PAGE and thereafter electro-transferred to PVDF membranes (Millipore, Bedford, MA). The membranes were blocked and incubated overnight at 4 °C with primary antibodies against N1ICD (1:1000), Hey1 (1:1000), Jagged-1 (1:1000), Hif-1α (1:1000), VEGFR2 (1:1000), and β-actin (1:5000) proteins. β-actin was used as an internal control. We cut the blots prior to hybridisation with antibodies according to the molecular weight of target protein. The membranes were rinsed using PBS and re-incubated with HRP-conjugated IgG (1:2000) antibodies and visualized using enhanced chemiluminescence (ECL) (Millipore, USA). The relative concentration of the aforementioned proteins were determined using scanning densitometry (ChemiDoc XRS1 Systems, Bio-Rad Laboratories, Inc.). Protein expression was analyzed with Image Lab 5.0 Software (Bio-Rad Laboratories, Inc.).

### Tubular formation assays

The difference in differentiation of MSCs was evaluated using reduced Matrigel Basement Membrane Matrix Growth Factor (Corning, NY, USA), following the manufacturer’s protocol. Briefly, Matrigel was first pre-warmed at 4 °C overnight before adding 50 μL/cm^2^ of the solution in 96 well-plates (Corning Incorporated, NY, USA). To solidify the Matrigel, we incubated the plates at 37 °C for 30 min. Induced or no-induced MSCs were differentiated for 14 days as described in the preceding sections, following the manufacturer’s protocols. Cells in all groups were first trypsinized before plating (2 × 10^4^ cells/cm^2^) in the Matrigel. The cells were examined and photographed under 10 × objective magnification after 24 h. The corresponding images/plates were analyzed using Image J angiogenesis analyzer. Parameters were set for images for basic network analysis; phase contrast images were analyzed for tube area and total tube length.

### Luciferase reporter assays

RBP-Jκ luciferase reporter vector (pGL-3) was purchased from Sangon Biotech, Shanghai. The vector was cloned with six repeated EBNA2RE sequences (GGTACCCGTGGGAAAATCGTGGGAAAATCGTGGGAAAATCGTGGGAAAATCGTGGGAAAATCGTGGGAAAATCTCGAGCTCGAG), using Kpnl and Xhol sites, before the firefly luciferase gene. EBNA2RE gene was removed to insert Neg-pGa981-6 before cloning into an irrelative fragment that cannot combine with RBP-Jκ. Transfection of MSCs was performed using 1 μg pGL-3 or neg-pGL-3 and 0.1 μg PRL-TK Renilla luciferase vector (10:1) (Promega, Madison, WI) using FuGene6 Transfection Reagent (Roche), following the manufacturer’s protocol. Cells were then seeded and cultured for 48 h in 24-well plates. The dual luciferase assay (Promega) was employed for the determination of Firefly and *Renilla* luciferase activities.

To determine whether N1ICD-induced Jagged1 upregulation can activate the Notch pathway in normal MSCs (untransfected), co-culture assays were performed. MSCs (2 × 10^5^) were pre-seeded for 24 h prior- transfection with 1 μg pGL-3 or neg-pGL-3 and 0.1 μg of PRL-TK *Renilla* luciferase vector; 24 h after transfection, pGL-3- or neg-pGL-3-transfected MSCs were co-cultured with Lenti-N1ICD-MSCs or transfected MSCs-CON with or without CSE. Luciferase activity was determined 48 h post-transfection using a dual luciferase assay system (Promega). *Renilla* luciferase activity was used as the internal control. All experiments were performed three times in triplicate.

### CSE preparation

CSE was prepared as previously described [20, 21]. Here, 400 mL of cigarette smoke (Da Qianmen, Shanghai, China), was drawn into a three-way cock 50-mL plastic syringe and mixed thoroughly with 20 mL DMEM/F12. Each cigarette contained 12 mg of tar and 2.5 mg of nicotine. One cigarette was used for preparing 20 mL of CSE, and the each CSE used not more than 30 min after preparation. The CSE solution was sieved through an aseptic 0.22-μm filter. The concentration of CSE used in the study was 2%.

### Statistical analysis

Data are expressed as mean ± standard deviation. Groups comparisons were done with ANOVA and two-tailed, paired t test. The data was analyzed using SPSS V. 24.0 for (SPSS Inc., Chicago, IL, USA). Statistical significance was set at *P* < 0.05.

## Results

### Isolation and identification of MSCs

MSCs were isolated using the plastic adherence method described previously [22]. Cells in passage 3 (P3) fibroblast-like, spindle-shaped morphology (Additional file 2: Fig. S1A). In adipogenic medium, lipid vacuole accumulation was stained by oil red O (Additional file 2: Fig. S1B) after 2 weeks. In osteogenic induction medium, the differentiation of MSCs into osteocytes was confirmed based on positive alizarin red S staining (Additional file 2: Fig. S1C). Also, all the isolated cells lacked CD34, CD45, CD80, and CD86, but more than 95% expressed CD29, CD90, CD73, and CD105, typical MSCs markers [23] (Additional file 2: Fig. S1D).

### Differentiation of MSCs into ECs

**Fig. 1:**
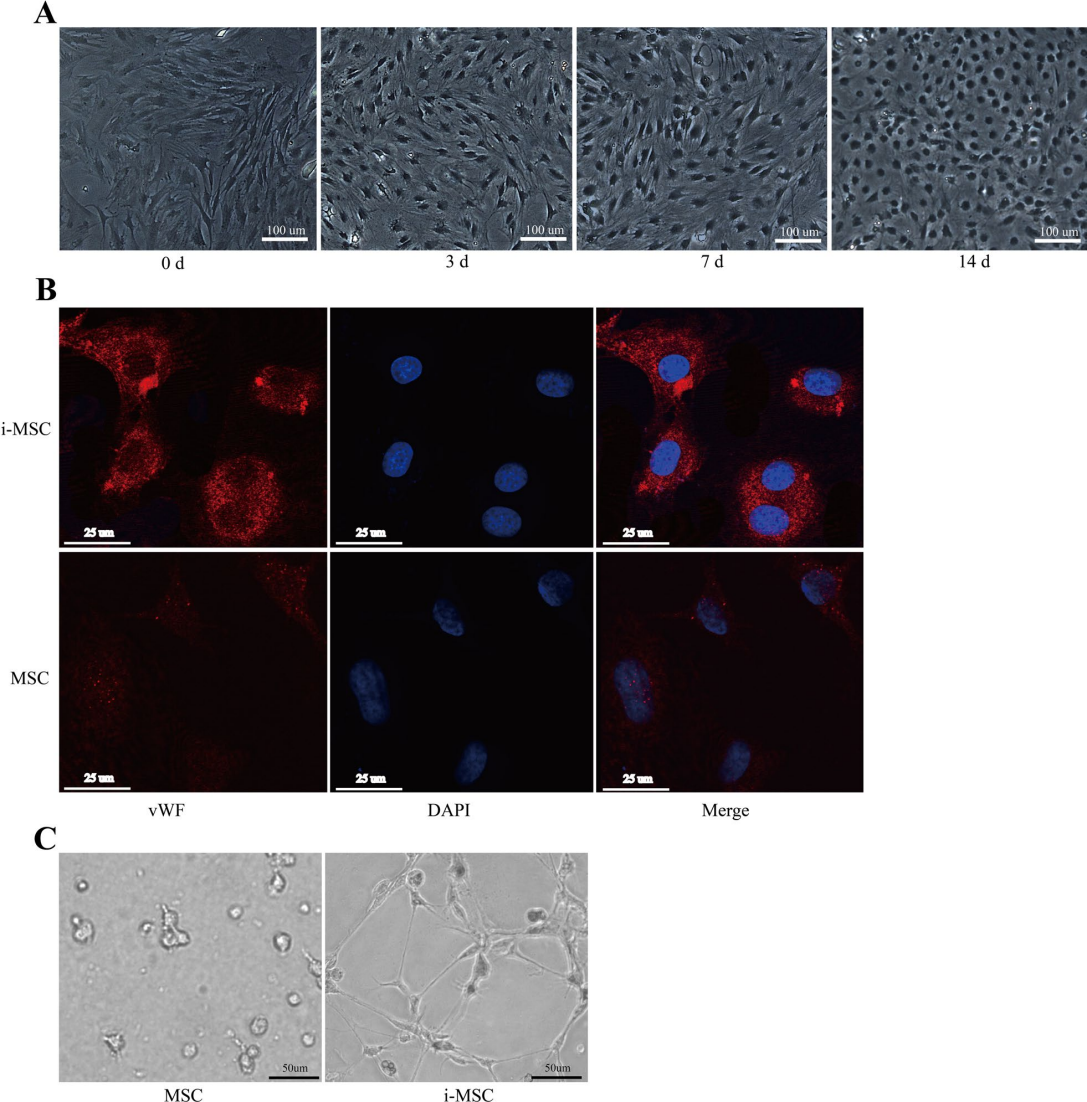
Dynamic changes in morphology, marker expression, and function of mesenchymal stem cells (MSCs) as they differentiate into endothelial-like cells after standard induction. **A** Induced MSCs retained a spindle-shaped morphology until day 3 and looked similar to rat aortic endothelial cells (ECs) after 14 days of induction (× 100). **B** vWF was detectable after 14 days of induction. However, we found only marginal expression of vWF in undifferentiated MSCs (× 400). **C** Induced MSCs showed tube-forming potential after culture in Matrigel for 24 h (× 200). i-MSC: induced MSC

First, we verified the feasibility of BM-MSCs differentiation into the ECs phenotype in defined medium in vitro. EGM-2 Bullet Kit medium supplemented with VEGF and bFGF at 10 and 2 ng/mL, respectively, was used as standard induction conditions. Notably, 2% ITS was used instead of FBS. Induced MSCs retained a spindle-shaped morphology until day 3 when cultured in standard induction conditions and exhibited detectable morphological changes after 14 days (Fig. 1A). The ECs [24] expressed the characteristic vWF marker at day 14 as shown in Fig. 1B. Finally, MSCs displayed their tube-forming potential in Matrigel 14 days after induction (Fig. 1C). This indicated that MSCs can differentiate into an ECs phenotype and form networks in standard induction conditions in vitro.

### Jagged1 expression is regulated by Notch signaling in MSCs

**Fig. 2:**
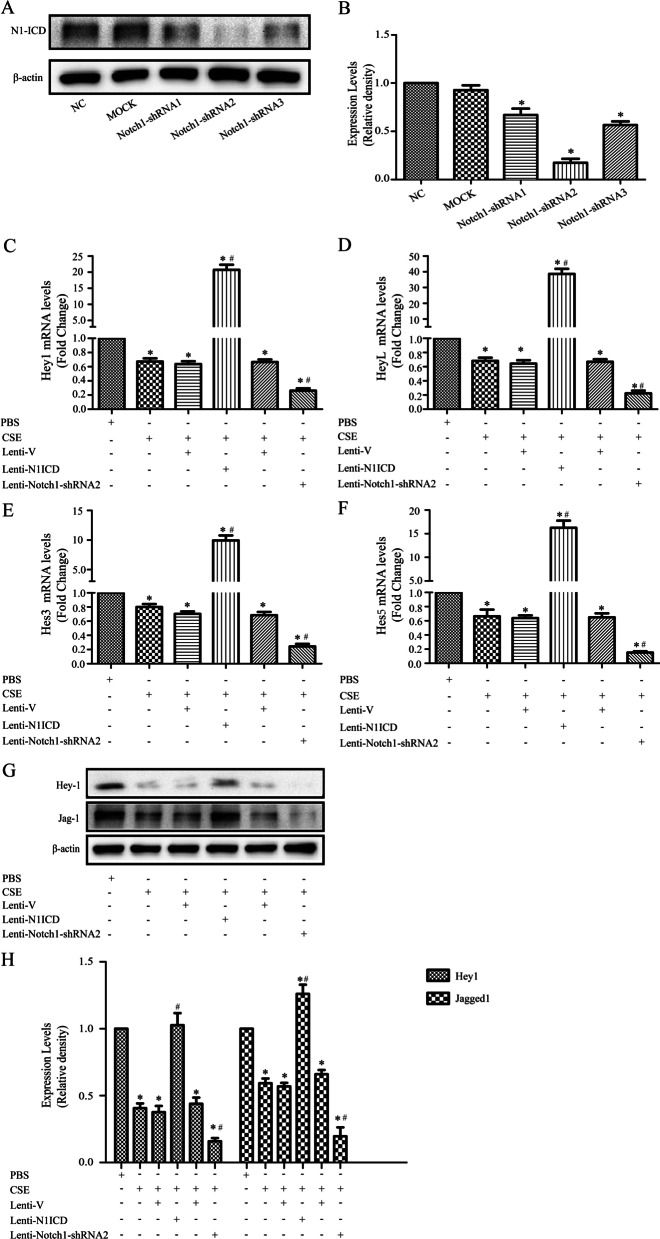
Jagged1 expression is regulated by Notch signaling in mesenchymal stem cells (MSCs). **A**, **B** Efficiency of Notch-1 knockdown in MSCs by lentiviral vectors harboring shRNA. MSCs were infected with lentiviral vectors GV248-Notch-1-shRNA1, 2, and 3 targeting Notch-1 or a scrambled shRNA vector (Mock) as detailed in Materials and Methods. Notch-1 protein expression was determined by western blotting assays. Notch-1 protein expression was normalized to β-actin levels. *P < 0.05 compared with non-infected MSCs. **C**–**F** The Notch signaling pathway was inhibited by incubation with CSE; N1ICD overexpression increased expression levels of Hey1, HeyL, Hes3, and Hes5, which were decreased after transfection with GV248-Notch-1-shRNA2. **G**, **H** CSE treatment downregulated Hey1 and Jagged1 expression levels; N1ICD overexpression rescued the decrease in MSCs, whereas the expression levels of Hey1 and Jagged1 were markedly decreased in GV-248-Notch1-shRNA2 transfected MSCs. *P < 0.05 vs. control group, #*P* < 0.05 vs. CSE group; CSE, cigarette smoke extract

First, we screened effective Notch1 shRNA. Three shRNAs were transfected into MSCs and the expression of N1ICD was tested 3 days post-transfection. Vehicle-treated MSCs were used as the mock group (Fig. 2A, B). A lentivirus overexpressing N1ICD independent of endogenous Notch1 signaling and analyzed Notch1 downstream target gene expression 3 days post-transfection was used to evaluate the effect of N1ICD on Notch signaling pathway. Compared to controls, over-expression of N1ICD up-regulated the expression of Notch1 target genes (Fig. 2C–F), indicating Notch activation.

Moreover, as shown in Fig. 2G, H, we found that activation of Notch signaling in MSCs up-regulated the expression of Hey1 and Jagged1. Interestingly, similar decreases in Jagged1 protein expression, as well as Notch1 downstream target genes, were also observed when Notch1 was inhibited by shRNA. These results are consistent with previous studies [25, 26] and suggest that Jagged1 is a downstream target of Notch1 signaling in MSCs.

### Role of Notch1 in MSCs differentiation into ECs

**Fig. 3:**
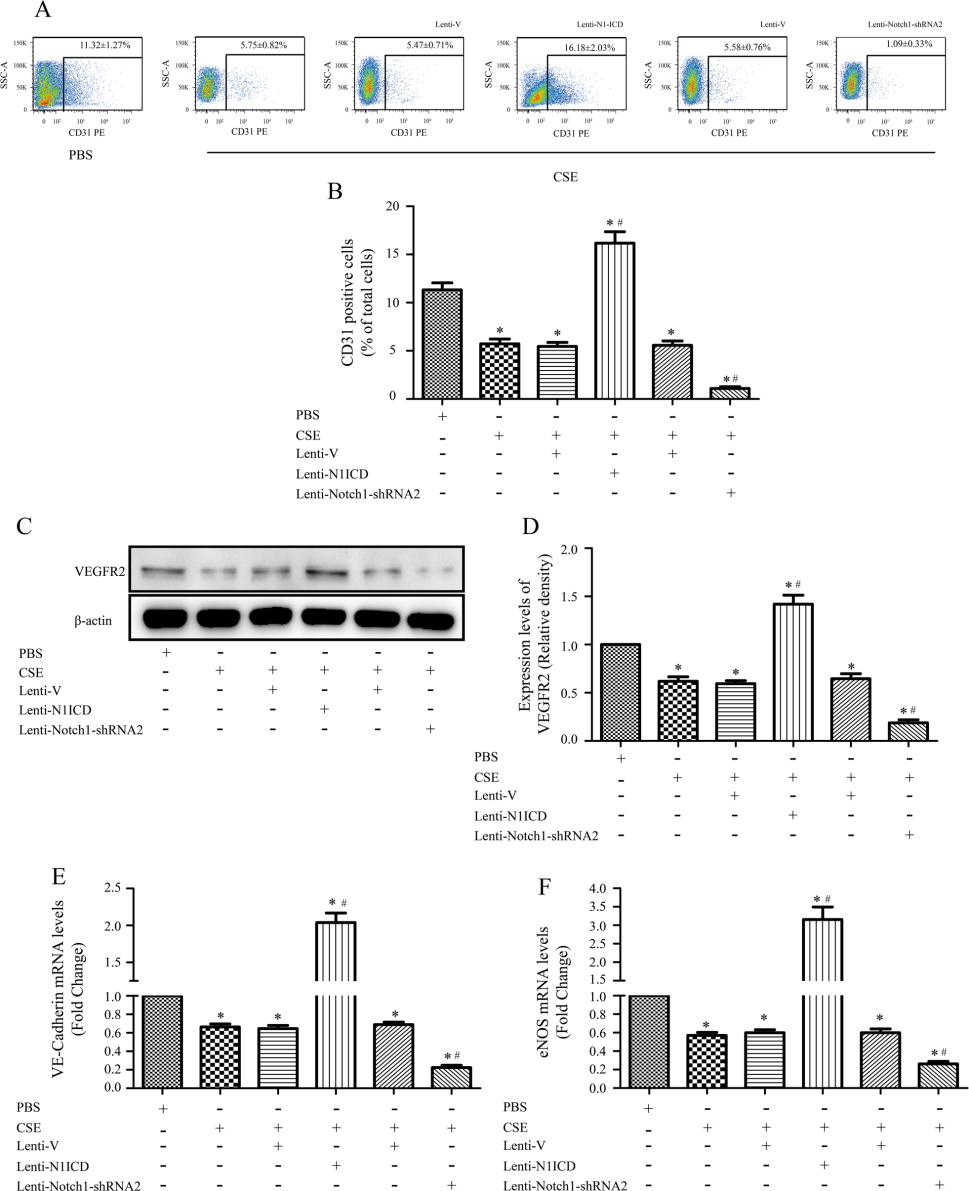
Regulation of Notch signaling induces endothelial cell (ECs) differentiation. **A**, **B** Versus that in the control group (11.32 ± 1.27%), the highest percentage of CD31 + cells (16.18 ± 2.03%) was observed in the N1ICD overexpression group; that in the CSE group was 5.75 ± 0.82% and that in the Notch-shRNA2 group was 1.09 ± 0.33%. **C**–**F** After 14 days of induction, VEGFR2 protein expression was determined by western blotting assays and eNOS and VE-cadherin transcript levels were determined by quantitative RT-PCR; N1ICD overexpression rescued the downregulation of VEGFR2, eNOS, and VE-cadherin expression induced by 2%CSE treatment, which were significantly decreased in GV-248-Notch1-shRNA2 transfected mesenchymal stem cells (MSCs). *P < 0.05 vs. control group, ^#^*P* < 0.05 vs. CSE group; CSE, cigarette smoke extract

To analyze the role of Notch1 signaling in ECs differentiation, differentiation experiments were performed for 14 days after induction. We found that approximately 11.32 ± 1.27% of cells in the normal, induced MSCs group were CD31+ . However, we found only marginal expression of CD31 in induced cells treated with 2%CSE (5.75 ± 0.82%). Furthermore, a significant reversal of CD31 positivity was observed when induced cells were transduced with N1ICD (16.18 ± 2.03%; Fig. 3A, B), suggesting that Notch1 abolishes the effects of CSE on MSCs differentiation into ECs. In addition, similar expression trends were observed for VEGFR2, VE-cadherin, and eNOS, which were decreased in the CSE treatment group and upregulated in N1ICD-LV cells compared to their expression in control groups (*P* < 0.05; Fig. 3C–F).

Furthermore, we treated MSCs with Notch shRNA after 14 days of differentiation and found that MSCs infected with Notch1 shRNA displayed significantly diminished CD31, VEGFR2, eNOS, and VE-cadherin expression compared to control levels (Fig. 3A–F), confirming that MSCs differentiation into ECs might depend on endogenous Notch signaling.

These data indicate that activation of N1ICD can rescue the inhibition of ECs differentiation induced by CSE and that endogenous Notch signaling regulates differentiation of MSCs into ECs.

### Notch signaling via RBP-Jκ promotes Jagged-1 expression

**Fig. 4:**
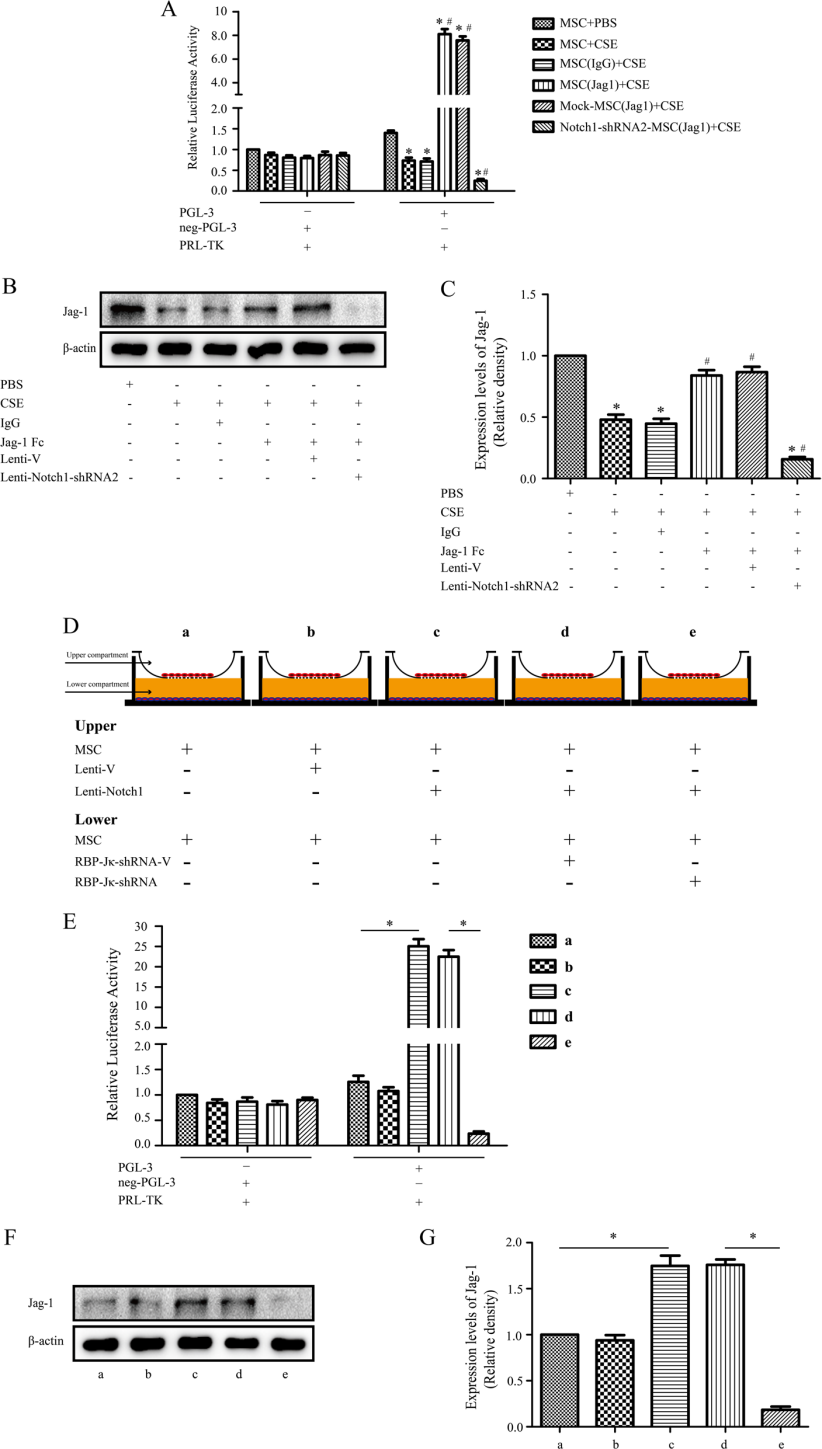
Notch signaling via RBP-Jκ promotes Jagged-1 expression. Jagged-1 enhanced the transcriptional activation of RBP- Jκ, which was endogenous Notch signaling-dependent. **A** 2%CSE treatment inhibited Notch1 reporter activation. Incubation with Jagged1 Fc significantly enhanced Notch1 reporter activation by greater than 8.1-fold relative to that in vehicle-treated mesenchymal stem cells (MSCs), whereas this improvement was suppressed by Notch-shRNA; Jagged1/Notch signaling also upregulated Jagged1 protein expression in MSCs and this response was blocked by Notch1-shRNA2 accordingly **B,** **C** *P < 0.05 vs. control group, ^#^*P* < 0.05 vs. CSE group; CSE, cigarette smoke extract. **D** MSCs, Lenti-V-MSCs, and N1ICD over-expressing MSCs were co-cultured with MSCs expressing a Notch reporter gene 24 h after transfection. **E** After 24 h of co-culture, Notch reporter expression was significantly increased in N1-ICD-overexpressing MSCs in the lower compartment relative to that in control co-cultures, whereas a significant decrease was observed when the lower compartment cells were pre-transfected with RBP-Jκ shRNA. **F**, **G** Jagged1 protein expression in lower compartment cells was determined by western blotting, and these cells exhibited similar expression trends with Notch reporter expression. *P < 0.05

In the non-canonical Notch pathway, Notch-dependent processes occur through RBP-Jκ-independent mechanisms [27]. To dissect the signaling pathways that mediate the effect of activated Notch on ECs differentiation, we used luciferase reporter assay to evaluate the effect of Jagged1 Fc on Notch signaling in MSCs. As shown in Fig. 4A, 2%CSE treatment inhibited Notch1 reporter activation. Incubation with Jagged1 Fc significantly enhanced Notch1 reporter activation, greater than 8.1-fold relative to that in vehicle-treated MSCs, whereas this effect was suppressed by Notch-shRNA, indicating that Jagged1 Fc enhances the transcriptional activation of RBP-Jκ, which is dependent on endogenous Notch signaling. Interestingly, Jagged1/Notch1 signaling also up-regulated Jagged1 expression in MSCs and this response was blocked by Notch1-shRNA (Fig. 4B, C). These findings suggest that Notch targets Jagged1 is the downstream processes in MSCs.

As previous results showed that Notch signaling upregulates Jagged1, we determined whether Jagged1 could promote ligand-induced Notch signaling activation. N1ICD-overexpressing MSCs were co-cultured with MSCs 24 h after Notch reporter transfection (Fig. 4D). Jagged1 expression up-regulated the expression of Notch by 25-folds (Fig. 4E), which was statistically significant (*P* < 0.05). When N1ICD overexpressing cells were co-cultured with MSCs transfected with Notch in the presence of RBP-Jκ shRNA, a significant decrease was observed in reporter expression and Jagged1 levels (Fig. 4F, G). These findings demonstrated that Notch-mediated Jagged1 upregulation is dependent on RBP-Jκ via a positive feedback loop.

### Jagged1-promoted ECs differentiation depends on endogenous Notch1 signaling

**Fig. 5:**
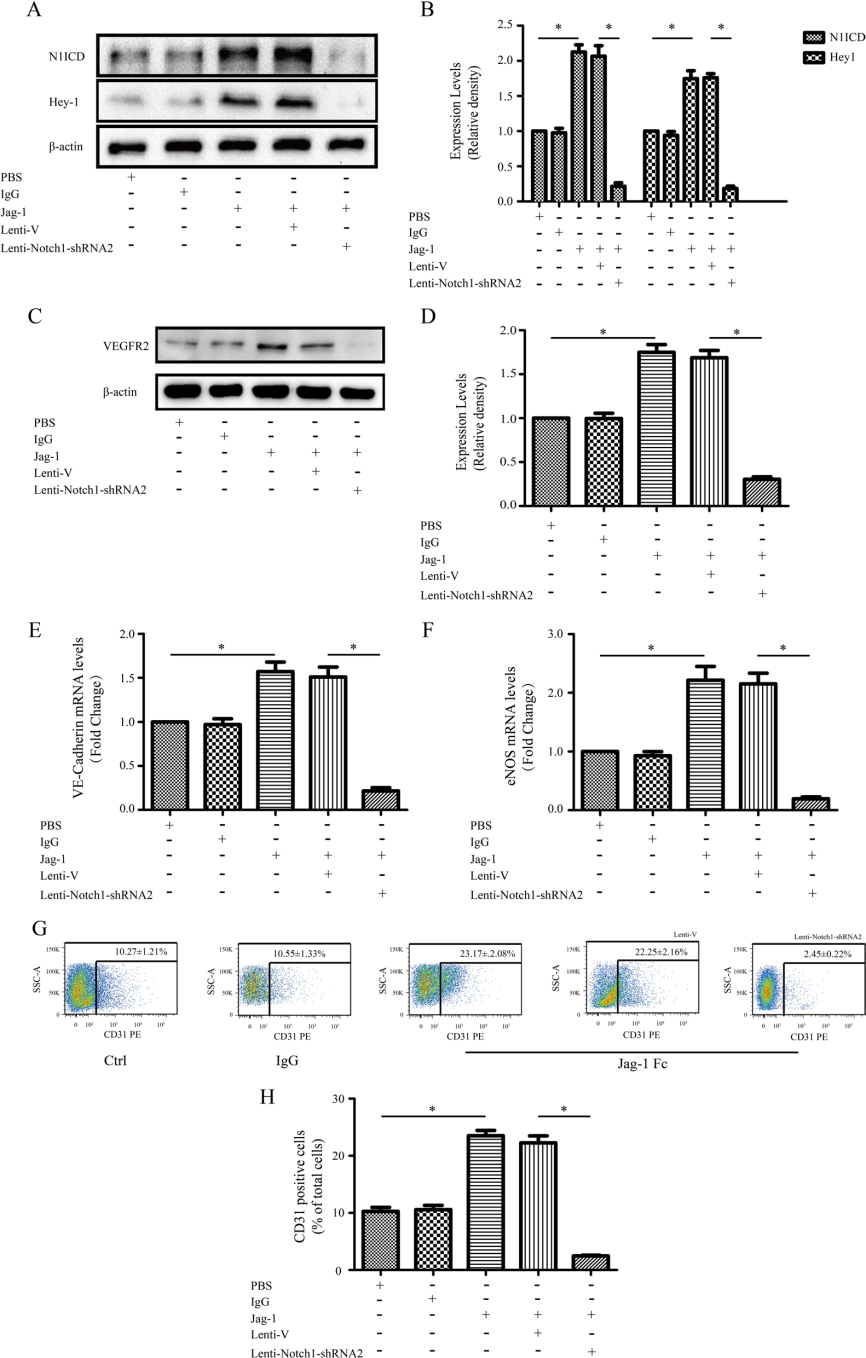
Jagged1 promotes endothelial cell (ECs) differentiation, which is dependent on endogenous Notch1 signaling. **A**, **B** Jagged1-Fc-activated Notch1 signaling in mesenchymal stem cells (MSCs), as shown by the upregulated protein expression of N1ICD and Hey1; this response was blocked by Notch1-shRNA2. **C**–**H** Jagged1 enhanced the expression levels of CD31, eNOS, VE-cadherin and VEGFR2; however, a significant reversal of this effect was observed when induced cells were transfected with Notch1-shRNA2 lentivirus. *P < 0.05

As the activated form of Notch1 promoted ECs differentiation, we wondered whether Notch1 could induce this phenotype as a result of ligand binding. To test this hypothesis, we used Jagged1 Fc to induce Notch1 signaling in MSCs, as indicated by increased luciferase signals, demonstrated previously herein. Further assays were performed to reveal the functions of endogenous Notch pathway in ECs differentiation by Jagged-1 Fc. Exogenous recombinant Jagged1-Fc activated Notch1 signaling in MSCs as evidenced by upregulation of N1ICD and Hey-1, and that this response was blocked by Notch1 shRNA. This activation was not apparent following culture with the IgG control (Fig. 5A, B). Furthermore, enhanced expression of CD31, eNOS, VE-cadherin and VEGFR2 was observed, indicating that ECs differentiation was promoted by Jagged-1 Fc (Fig. 5C–H). However, a significant reversal of this effect, as evidenced by decreased expression of ECs-specific markers, was observed when induced cells were transduced with Notch1 shRNA. These findings demonstrated that Jagged1-Fc fragments trigger Notch1 signaling to promote MSCs differentiation into ECs lineages and the observed activation is necessary for inducing endogenous Notch signaling.

### Notch1 signaling promotes vessel assembly

Notch1 is associated with several transcription factors such as HIF-1α [28], which participates in angiogenesis. To validate the role of Notch1 signaling in angiogenesis, we overexpressed N1ICD in MSCs for 14 days and assessed vessel assembly in Matrigel-coated plates.

**Fig. 6:**
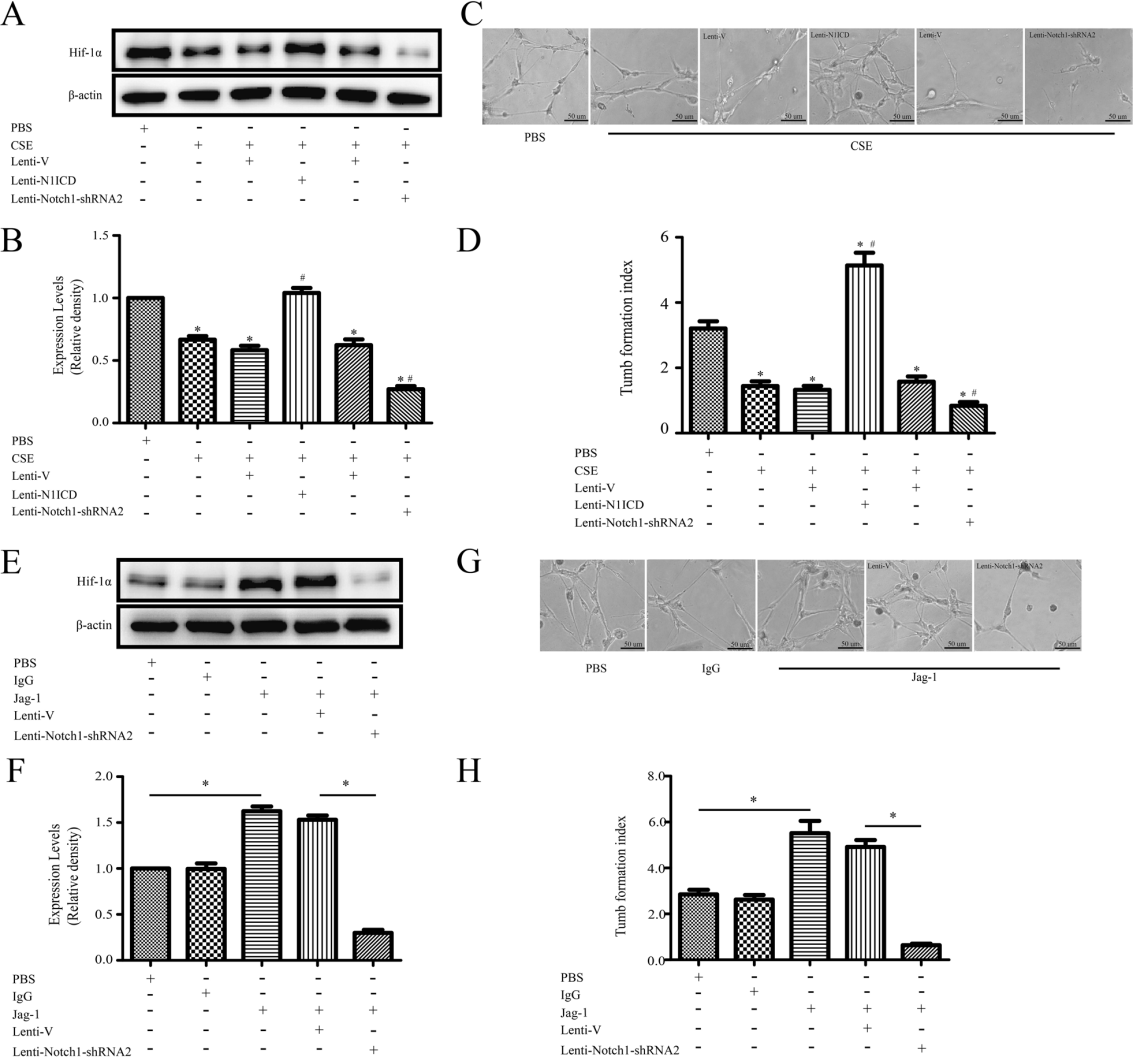
Notch1 signaling promotes vessel assembly. **A,** **B** Hif-1α protein expression levels were increased in N1ICD-MSCs (mesenchymal stem cells), but were decreased in the 2%CSE group. A statistically significant difference in vessel assembly capacity, as evidenced by the number of tubule-like structures, was also found between cultures of N1ICD MSCs and the CSE-treatment group (**C**, **D**). MSCs infected with Notch shRNA2 displayed significantly reduced protein levels of Hif-1α, as well as vessel assembly capacity, compared to those in the CSE treatment group, *P < 0.05 vs. control group, ^#^*P* < 0.05 vs. CSE group; CSE, cigarette smoke extract. **E**–**H** Treatment with Jagged1 Fc improved the expression of HIF-1α, as well as vessel assembly capacity, whereas Notch1 shRNA2 blocked the promotion of vessel assembly induced by Jagged1, as shown by a significant reversal in the number of tubule-like structures and the protein expression of Hif-1α. *P < 0.05

First, we determined whether Notch1 expression could upregulate HIF-1α; we found N1ICD upregulated Hif-1α protein level in MSCs but 2%CSE decreased Hif-1α protein (Fig. 6A, B). N1ICD also influenced tubule-formation properties of MSCs, relative to controls (*P* < 0.001), indicating that Notch1 signaling could reverse the inhibitory effect of CSE on vessel assembly (Fig. 6C, D). Furthermore, to determine the role of endogenous Notch pathway in vessel assembly, we treated MSCs with Notch1 shRNA and cultured them in standard induction medium. Vehicle-treated MSCs were used as a control. We observed that MSCs infected with Notch shRNA displayed significantly reduced vessel assembly capacity, relative to controls, as evidenced by the tube formation index.

Moreover, co-culture with Jagged1 Fc could not rescue the decreased expression of HIF-1α induced by Notch shRNA, even though Jagged1 significantly increased the protein levels of Hif-1α (Fig. 6E, F). Tube formation assays also supported the findings that Jagged1-mediated vessel assembly is Notch1-dependent (Fig. 6G, H). These observations demonstrate that expression of HIF-1α is regulated by the Notch1 pathway, emphasizing that Jagged1/Notch1 signaling might function through the upregulation of HIF-1α to increase vessel assembly.

## Discussion

Herein, we explored the role of Jagged-1/Notch1 signaling on differentiation of endothelial MSCs. This is the pioneer study on exploring mechanism underlying differentiation of MSCs into ECs. These data suggest that Jagged-1/Notch1 signaling promotes ECs differentiation from MSCs and provide an effective and viable way to generate therapeutically-relevant numbers of ECs. Repair and/or regeneration of damaged endothelia could conceivably maintain alveolar structures and prevent emphysema. MSCs possess inherent property to differentiate in to numerous functional cell types that can repair diseased or injured tissue. Over the last two decades, there has been significant progress in the field of regenerative medicine and stem cell technology [29]; however, the efficiency of re-endothelialization and sub-sequent clinical outcomes vary considerably [30]. The aim of this study was consistent with previous study [9], which dedicated to improving the efficiency of MSCs differentiation ECs in vitro for potential application in the treatment of peripheral arterial disease. Furthermore, in this study, we investigated mechanism underlying differentiation of MSCs into ECs. Notch signaling can fine-tune differentiation in cellular fields during morphogenesis [31]. Emerging evidence has demonstrated that Notch signaling pathway participates in the differentiation, proliferation and apoptosis of cells. Jiang et al. found that activation of Notch signal pathway can promote Th2 cell differentiation in acute exacerbation of COPD [32]. Mice models have demonstrated that inhibiting Notch1 and Notch4 impaired angiogenesis in ECs [15], resulting in part from disrupted differentiation of mesodermal and endodermal progenitors into vascular ECs. Moreover, Notch1 has been shown to promote angiogenesis in ECs, underlining the role Notch1 pathway in angiogenesis [28]. Previous study showed that Jagged1 ligand was found to be the only Notch ligand shuttled into MSCs-derived exosomes [33]. We hypothesize that Jagged1 activates differentiation of MSCs by binding Notch receptors. Notch activation in MSCs triggers ECs differentiation, resulting in up-regulates the expression of ECs markers and promotes tube formation properties of the ECs, and simultaneously directly activates Jagged1 expression, which is dependent on RBP-Jκ. This positive feedback progressively activates Notch in adjacent cells, further promoting differentiation of ECs. These events combine to promote angiogenesis. Eventually, the signal weakens, or inhibited by antagonist morphogens that are yet to be identified. Indeed, this theory has been backed by empirical experimental data. Expression of Jagged1 in the endothelium is necessary for differentiation of adjacent mesenchymal cells [34]. Research shows that inhibition of Notch impairs expression of Jagged1 both in vivo in vitro [25]. Jagged1 as a Notch target is in agreement with published data that supports positive feedback between Notch and Jagged1. For instance, NIH 3T3 cells activates expression of Jagged1 via Notch signaling pathway [35]. Moreover, shRNA-mediated knock down of either Notch or RBP-Jκ in macrophages down-regulates the expression of Jagged1 [36]. Notch activation in the basal epidermis corresponds with over-expression of Notch and Jagged1 both in the epidermis and dermis [37]. Finally, Feng et al. proposed a lateral induction mechanism involving Notch and Jagged1 to explain patent ductus arteriosus in mice lacking jagged1 in smooth muscle [38].

Zong et al. showed that overexpression of Notch1 with N1ICD in human pulmonary microvascular endothelial cells significantly alleviated the cell apoptosis induced by CSE, and concluded that Notch1 protects against CS-induced endothelial apoptosis in COPD through inhibiting the ERK pathway [39]. The preceding work showed that overexpression of N1ICD increased the expression of angiogenic factors and tube-formation capacity, correlating with improved ECs differentiation. Thus, we inferred that Notch1 worked in combination with other signaling pathways to promote angiogenesis. In this context, one previous study demonstrated that elevated HIF-1α in MSCs promoted angiogenesis [40]. In a more recent study, elevated HIF-1α in MSCs increased exosome secretion, which promoted angiogenesis in part by increasing packaging of Jagged1 [41]. Recent studies have demonstrated the association between Notch signaling pathway and expression of HIF-1α at different levels; For example, Soares et al. reported that expression of either an active or full-length form of Notch1 resulted in the upregulation of HIF-1α [28]. Furthermore, Saravana and colleagues demonstrated that Notch signaling promoted ECs angiogenesis and upregulated HIF-1α [42]. Overexpression of Notch1 facilitated angiogenesis of trophoblast cells while HIF-1α inhibitor suppressed [43]. Theasaponin E1 inhibited platinum-resistant ovarian cancer cells through activating apoptosis and suppressing angiogenesis via downregulating protein expression of Jagged1-Notch1-HIF-1α axis [44]. Therefore, based on our research results, we inferred that HIF-1α was a putative target of activated Notch1, suggesting Notch1/Jagged1 patriated in angiogenesis in a paracrine manner, in particular by upregulating the expression of HIF-1α.

From our understanding of stem cell biology, Notch1-mediated regulation of differentiation of MSCs into ECs has potentially far-reaching implications. In the 1950s, Liebow reported that alveolar septa in COPD patients were almost avascular [45]. Accordingly, it was thought that vascular atrophy damages alveoli and then the formation of emphysema, which is directly related to lung function. Therefore, restoring, or regenerating ECs might be important to repair lung function in patients with COPD/emphysema. Unfortunately, we have not yet determined whether Notch1 influenced the differentiation of MSCs into ECs in the absence of CSE stimulation, and whether differentiated ECs are able to transdifferentiate into MSCs, confirmation of this requires further research.

## Conclusions

We demonstrated the novel finding that Jagged-1/Notch pathway regulated differentiation of MSCs into ECs phenotypes, and this process was dependent on RBP-Jκ. Understanding how Notch1 signaling modulates these processes might provide new therapeutic strategies for COPD. Even so, more studies will be necessary to address these mechanisms and identify mediators of MSCs differentiation in vivo.

## Supplementary Information

**Additional file 1**. Original western blot images in the text.

**Additional file 2**. MSCs characterization.

**Additional file 3**. The original western blot images of β-actin.

## Data Availability

The datasets used and/or analysed during the current study available from the corresponding author on reasonable request.

## Abbreviations

bFGF: Basic fibroblast growth factor
COPD: Chronic obstructive pulmonary disease
CSE: Cigarette smoke extract
ECs: Endothelial cells
FBS: Fetal bovine serum
GAPDH: Glyceraldehyde-3-phosphate dehydrogenase
MSCs: Mesenchymal stem cells
NICD: Notch intracellular domain
PMBF: Pulmonary microvascular blood flow
PVDF: Polyvinylidene difluoride
VEGF: Vascular endothelial growth factor
vWF: Von Willebrand factor
