# Supplementary material for: Notch1 activation of Jagged1 contributes to differentiation of mesenchymal stem cells into endothelial cells under cigarette smoke extract exposure
Source: BMC Pulm Med. 2022 Apr 11;22:139. doi: 10.1186/s12890-022-01913-3 (PMC9004089; doi:10.1186/s12890-022-01913-3)
Supplement: Supplementary file 1 — Additional file 1. Original western blot images in the text. [file 12890_2022_1913_MOESM1_ESM.docx]

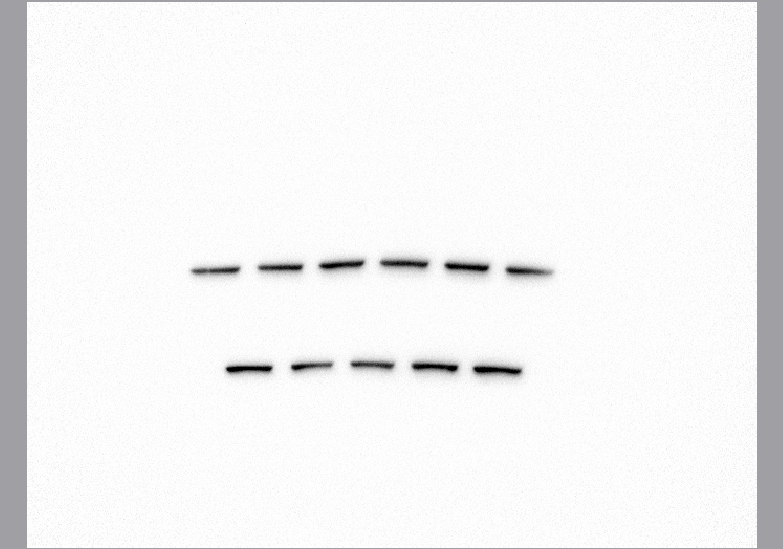

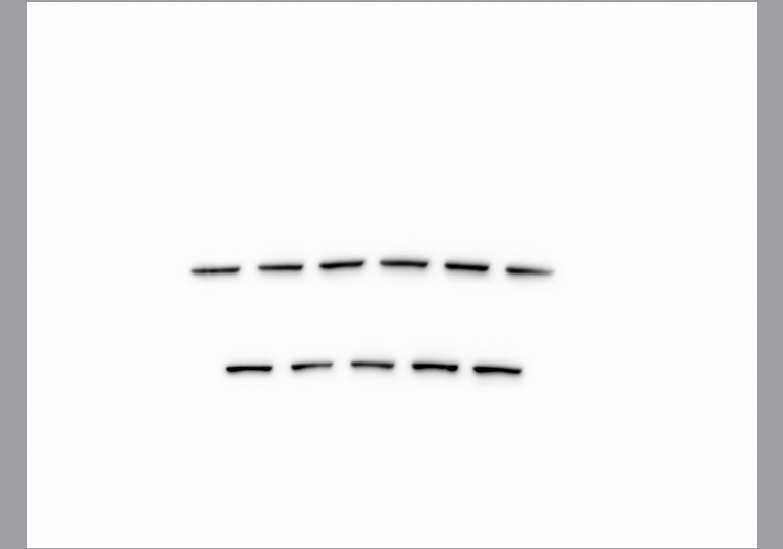

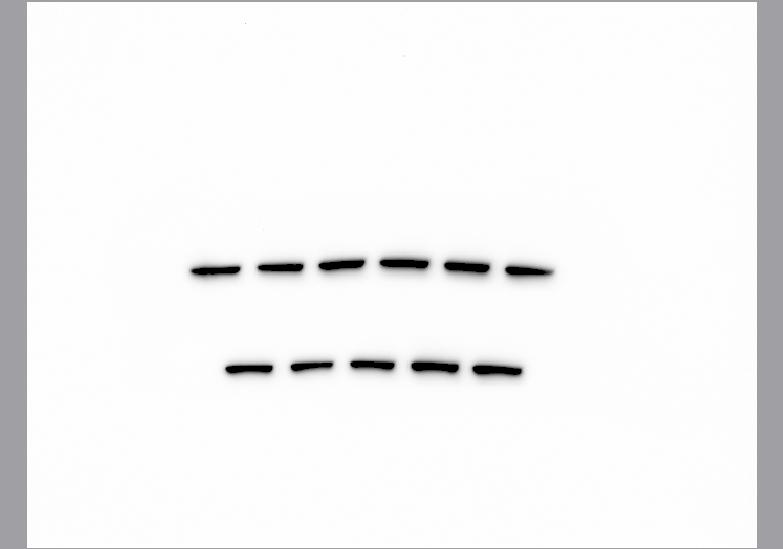

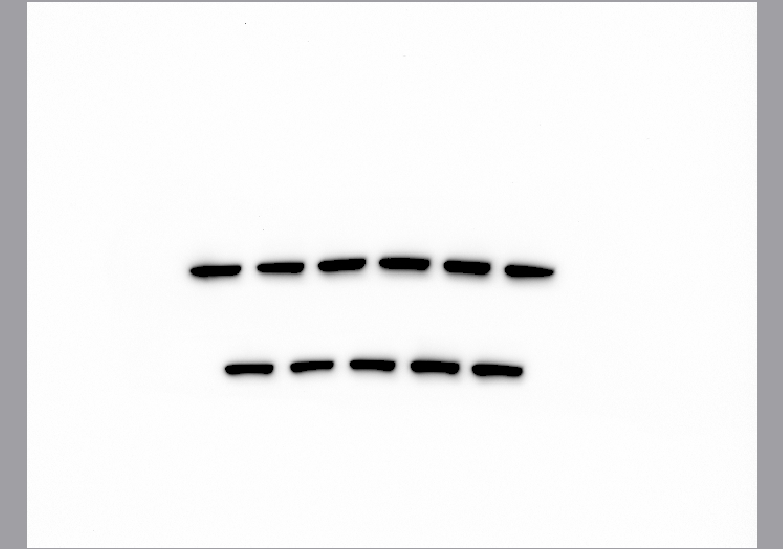

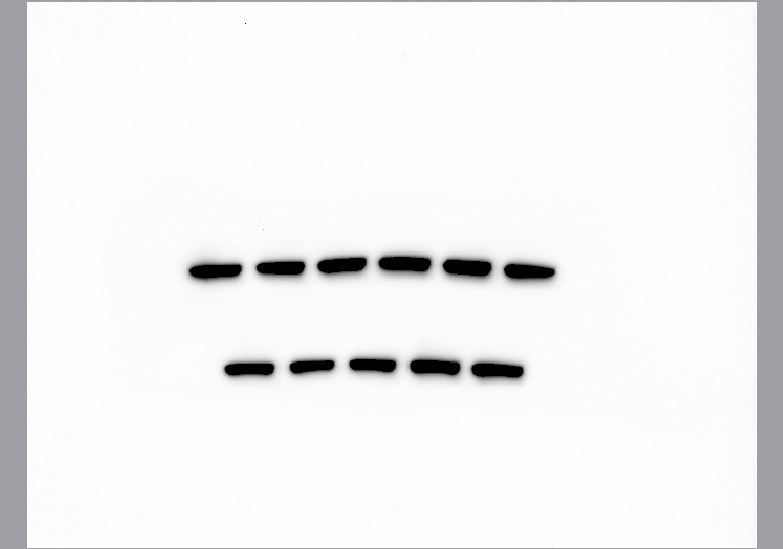


Fig2 A

β-actin

Fig2 A

β-actin

Fig2 A

β-actin

Fig2 A

β-actin

Fig2 A

β-actin

1. The bands below were the multiple exposure images of β-actin in Figure 2A of the text.


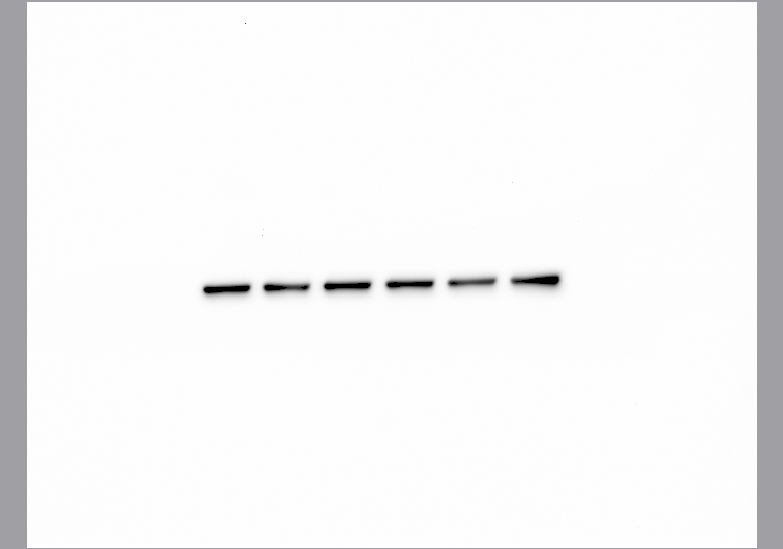

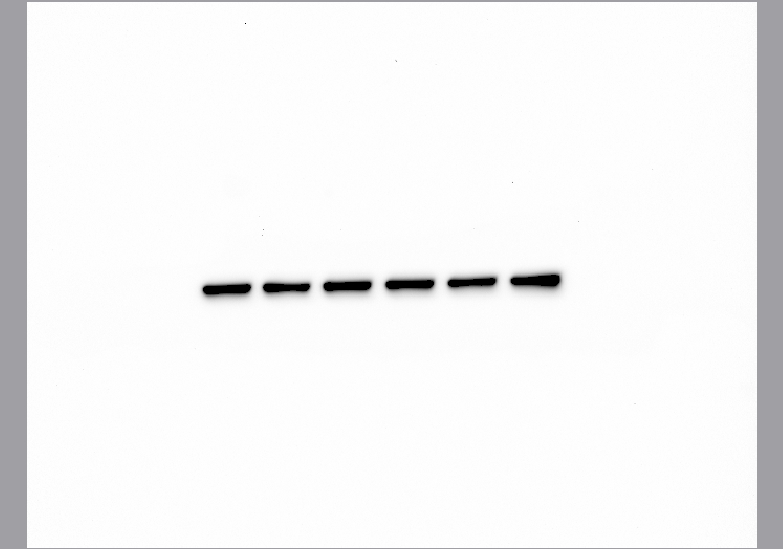

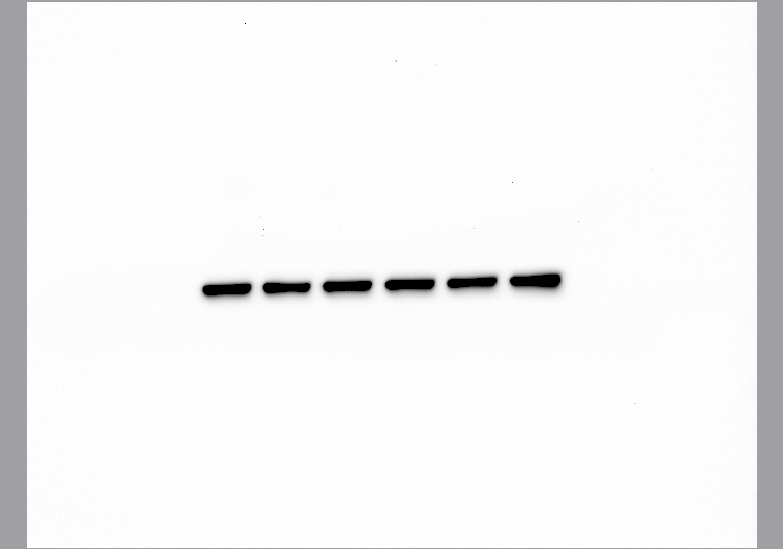


Fig 2G

β-actin

Fig 2G

β-actin

Fig 2G

β-actin

1. The multiple exposure images of β-actin in Figure 2G of the text.


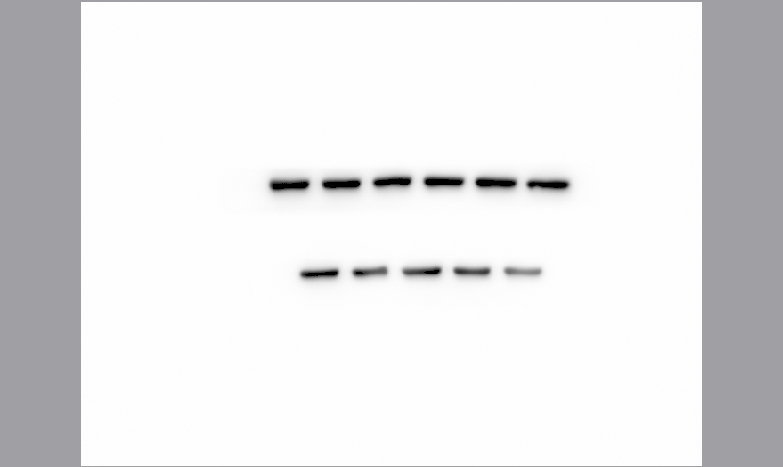

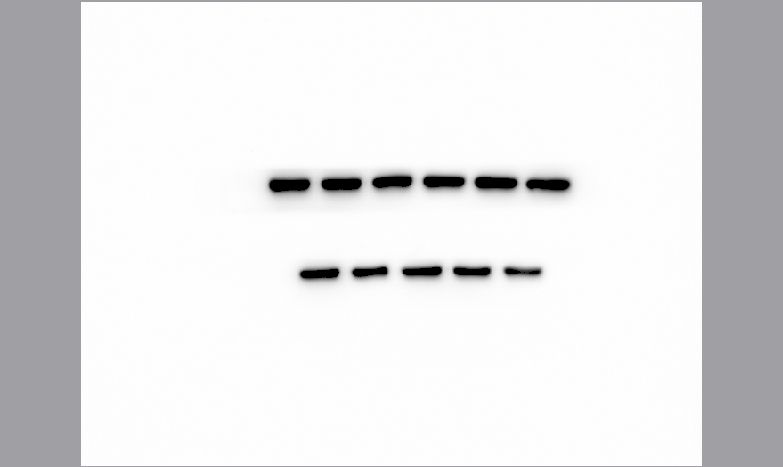

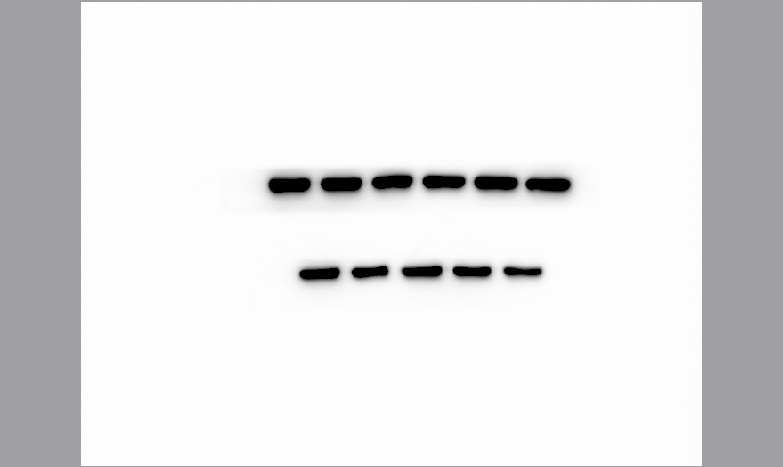

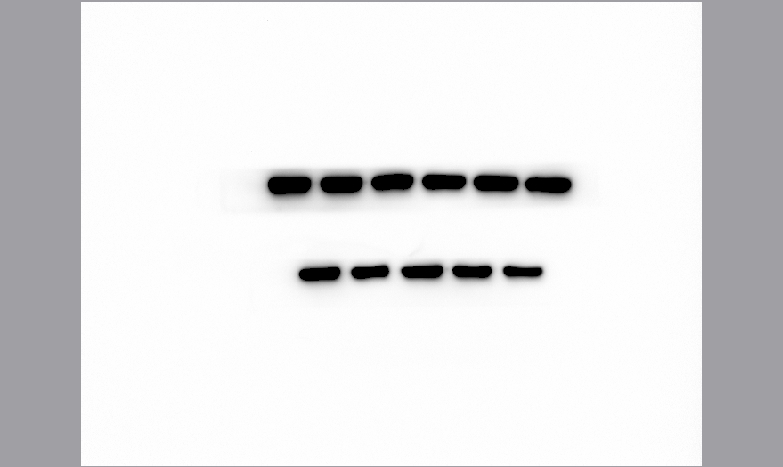

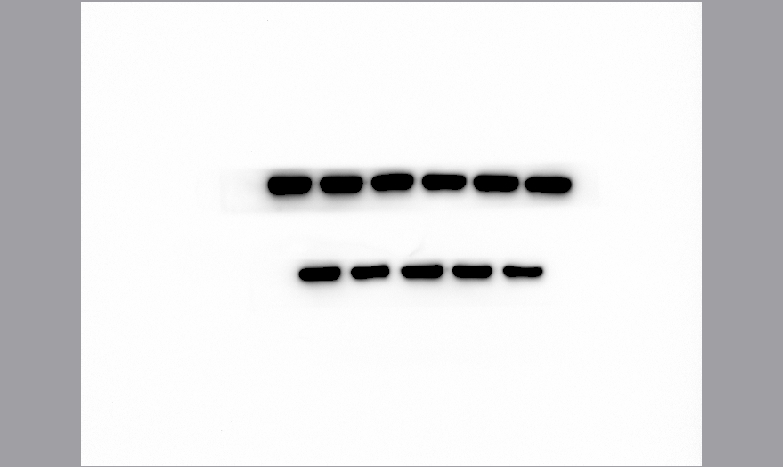

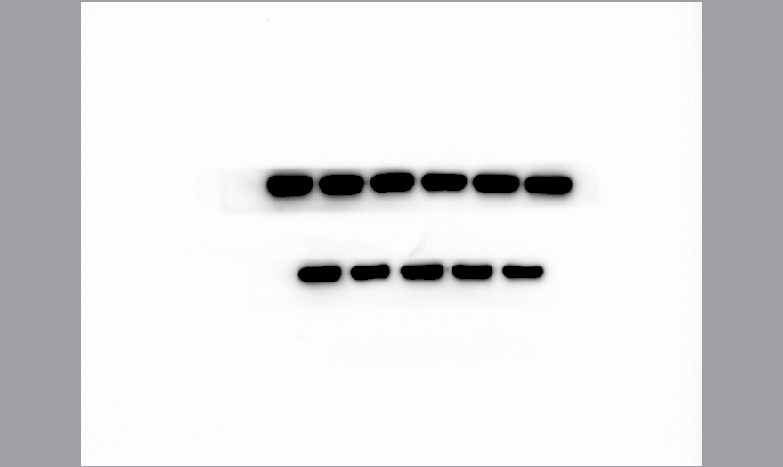


Fig 3C

β-actin

Fig 3C

β-actin

Fig 3C

β-actin

Fig 3C

β-actin

Fig 3C

β-actin

Fig 3C

β-actin

1. The bands above were the multiple exposure images of β-actin in Figure 3C of the text.


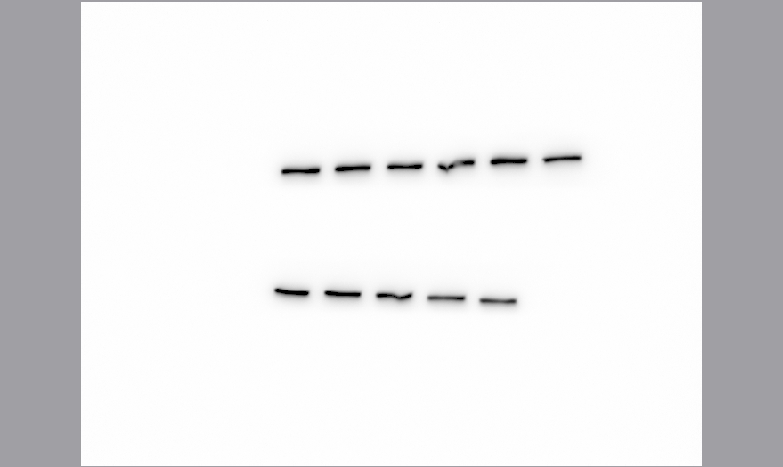

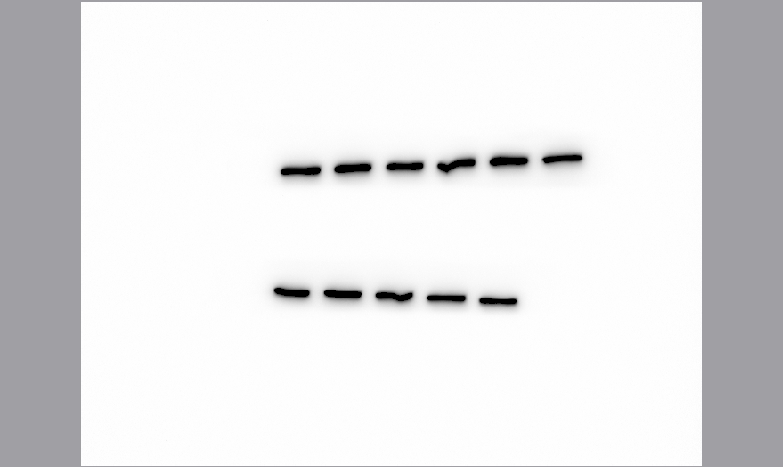

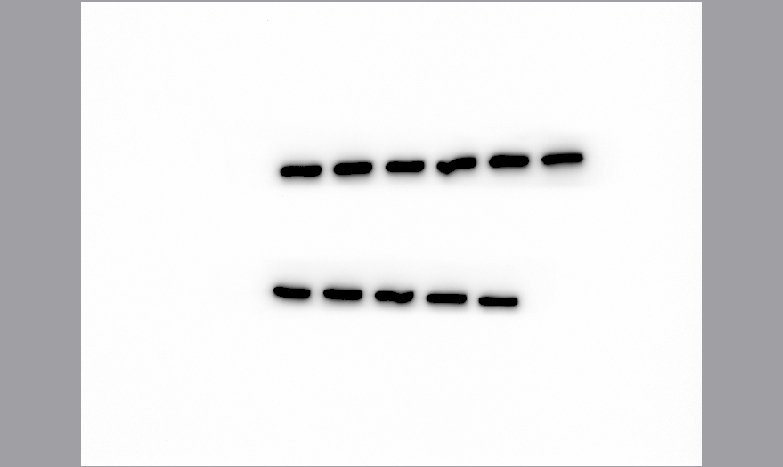

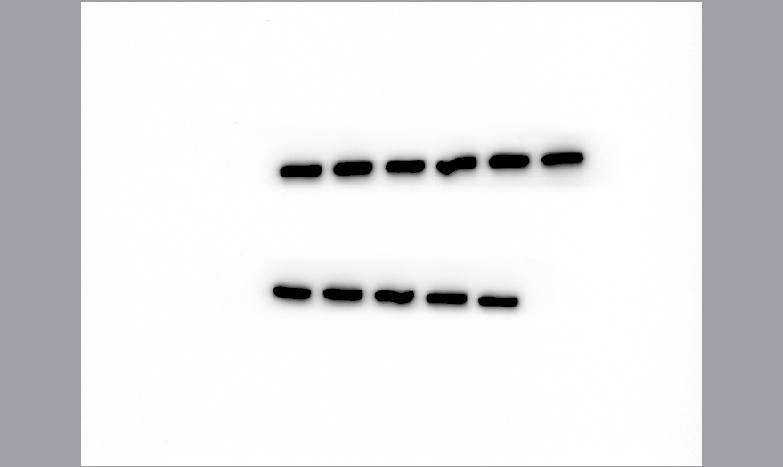

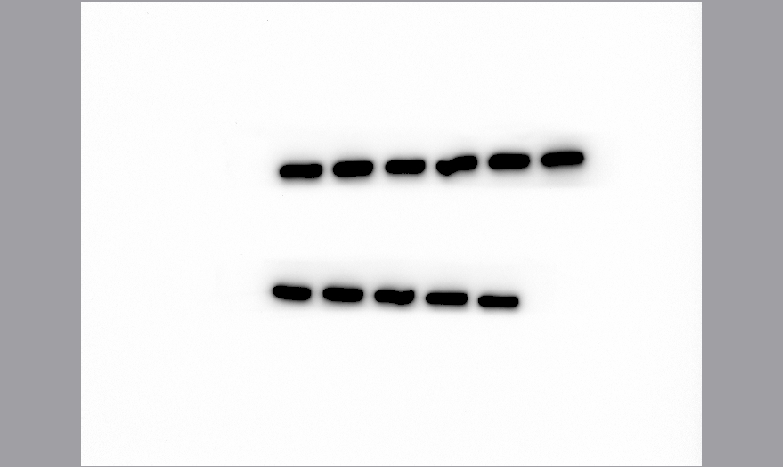


Fig 4B

β-actin

Fig 4B

β-actin

Fig 4B

β-actin

Fig 4B

β-actin

Fig 4B

β-actin

1. The bands above were the multiple exposure images of β-actin in Figure 4B of the text.


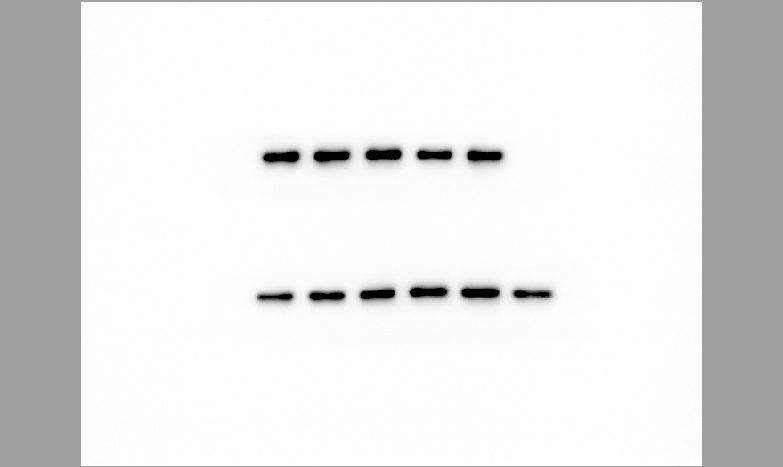

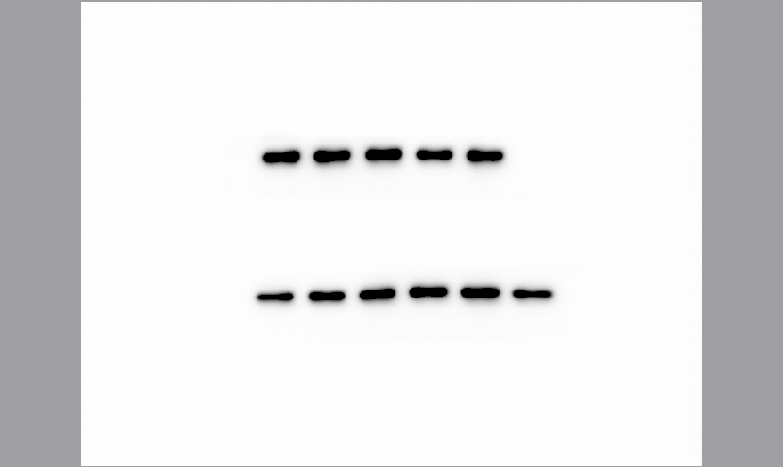

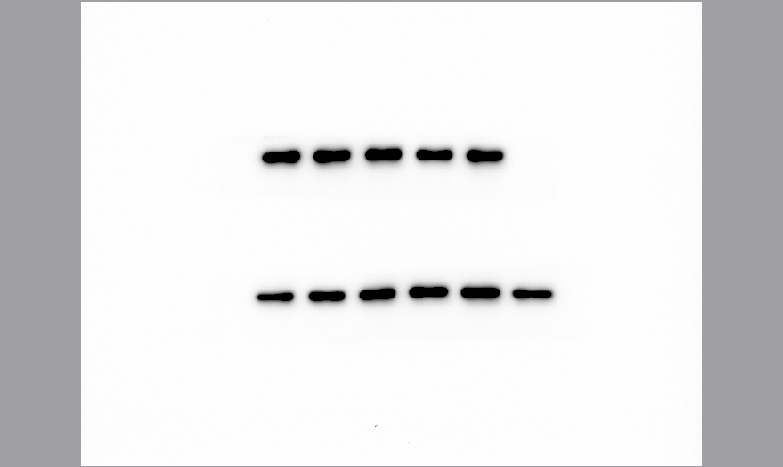

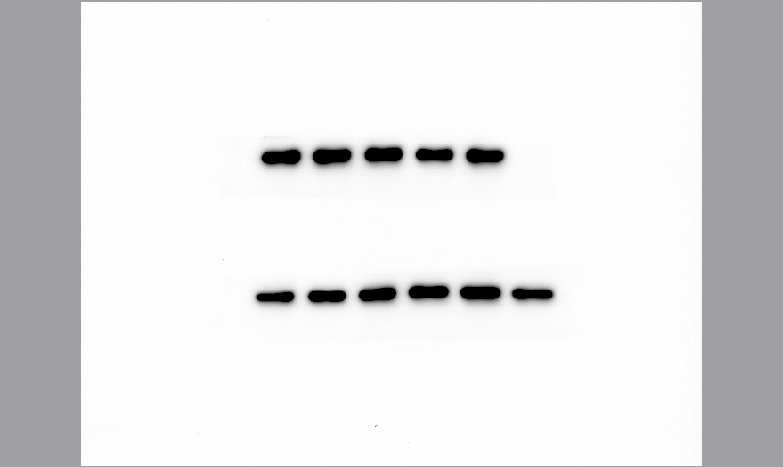

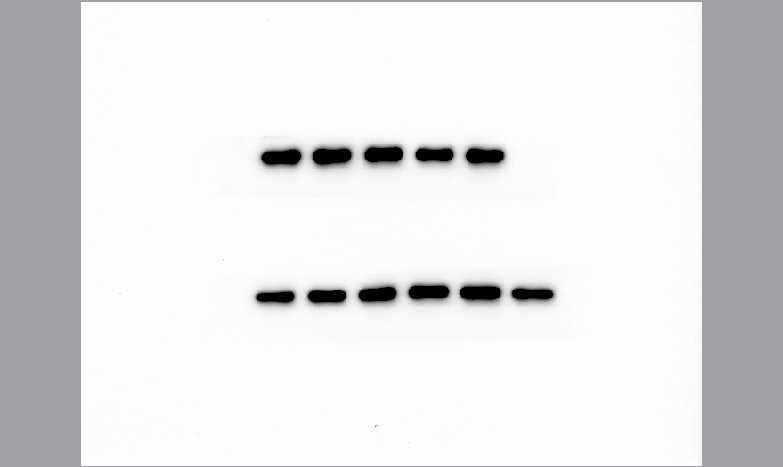

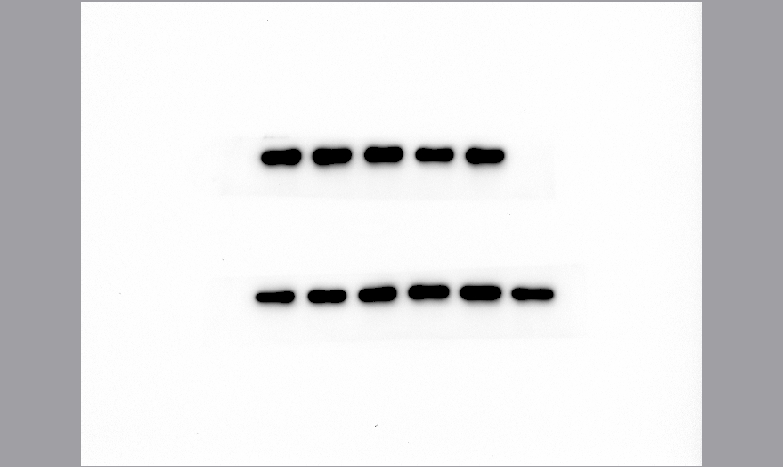


Fig 4F

β-actin

Fig 4F

β-actin

Fig 4F

β-actin

Fig 4F

β-actin

Fig 4F

β-actin

Fig 4F

β-actin

1. The bands above were the multiple exposure images of β-actin in Figure 4F of the text.


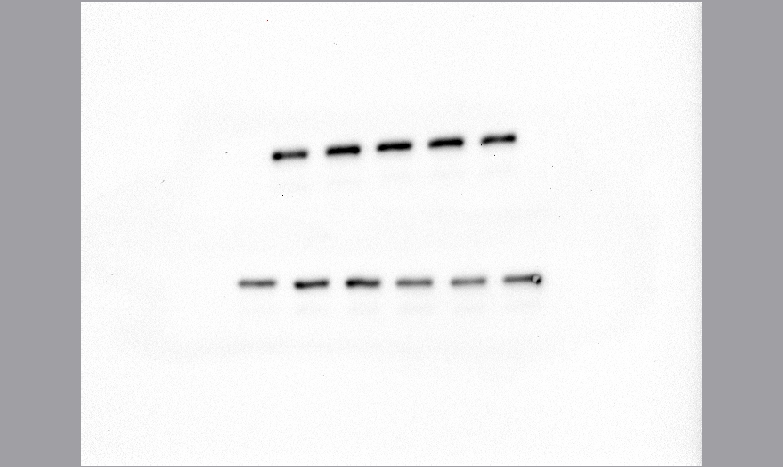


Fig 5A

β-actin


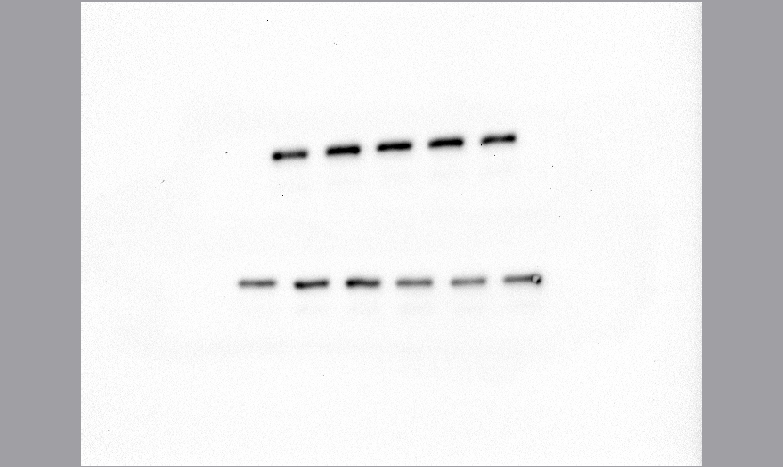


Fig 5A

β-actin


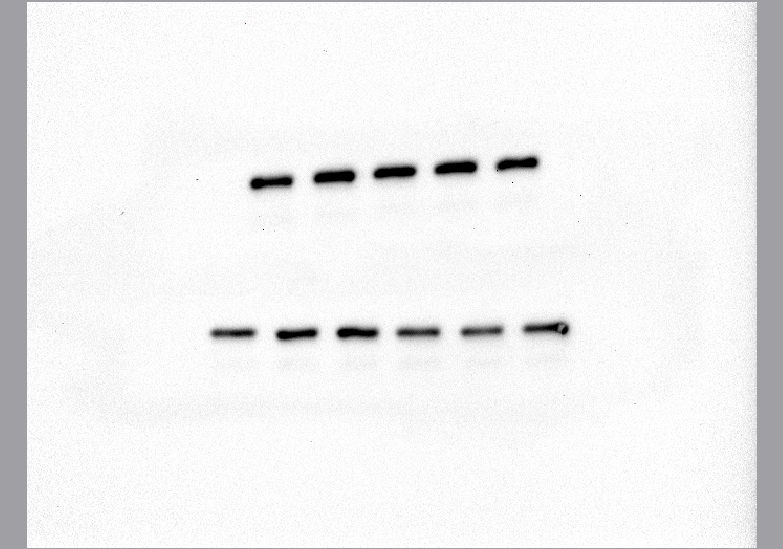


Fig 5A

β-actin

1. The bands above were the multiple exposure images of β-actin in Figure5A of the text.


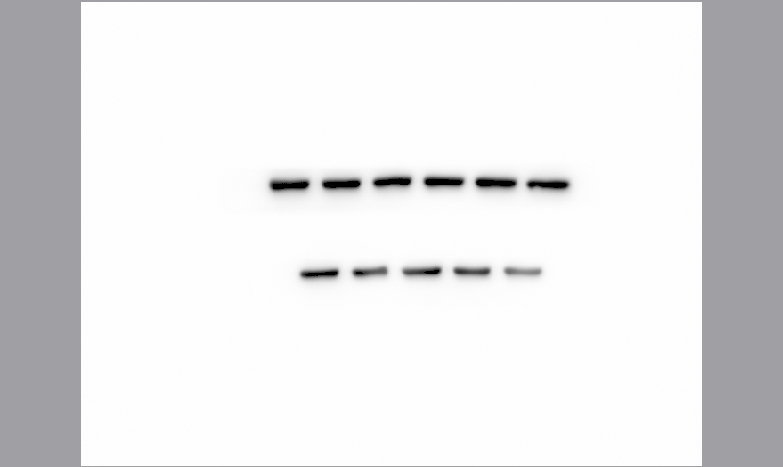

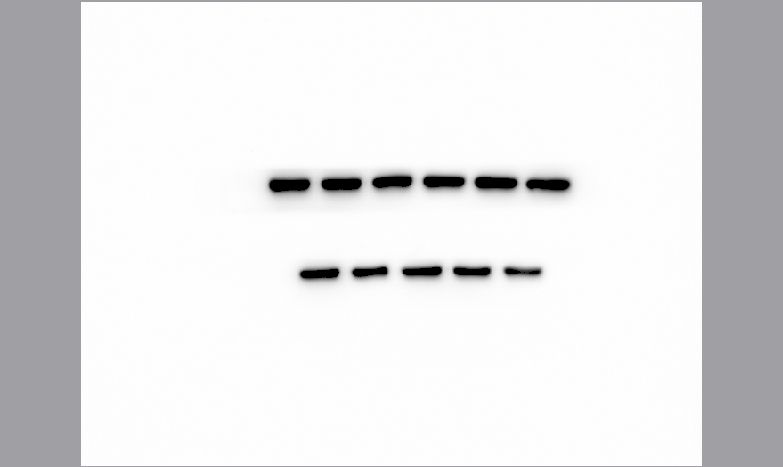

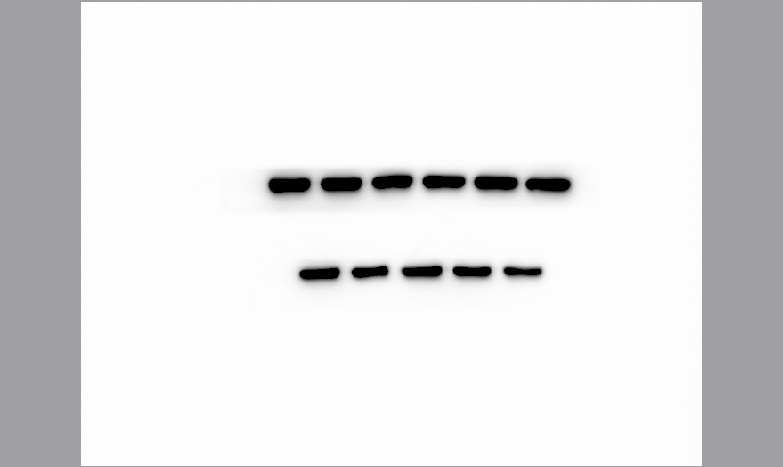

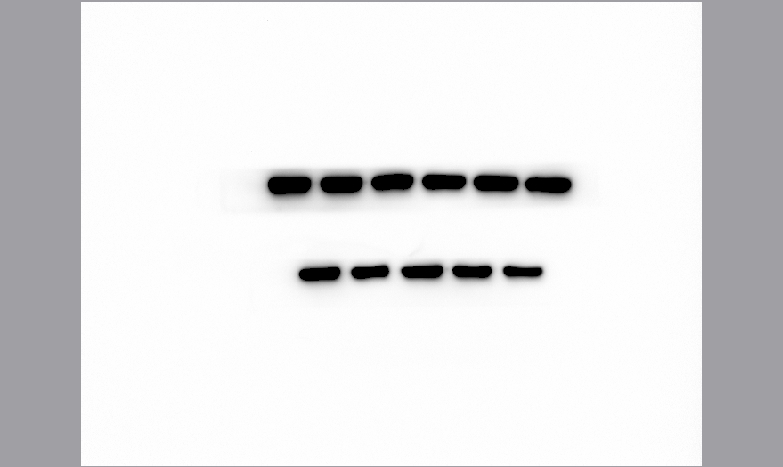

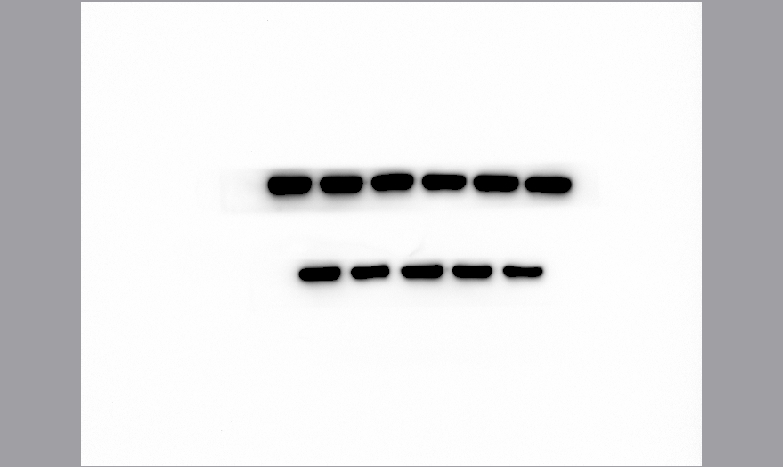

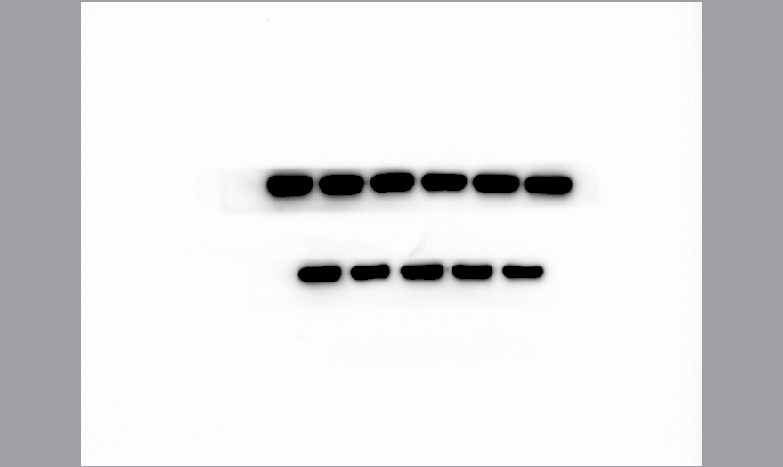


Fig 5C

β-actin

Fig 5C

β-actin

Fig 5C

β-actin

Fig 5C

β-actin

Fig 5C

β-actin

Fig 5C

β-actin

1. The bands below were the multiple exposure images of β-actin in Figure 5C of the text.


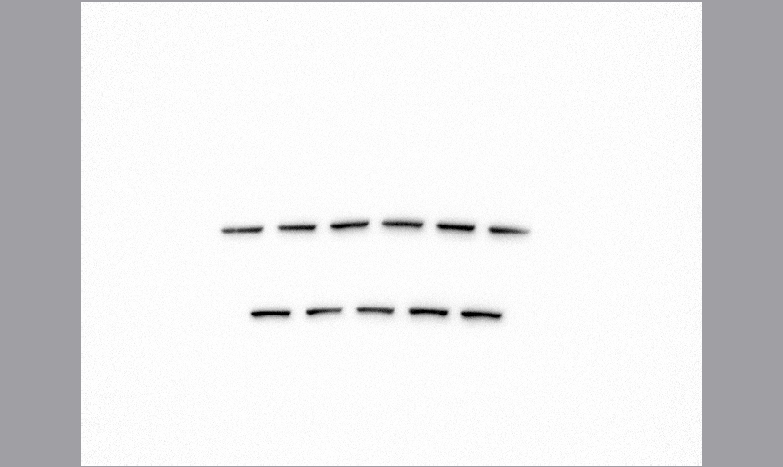

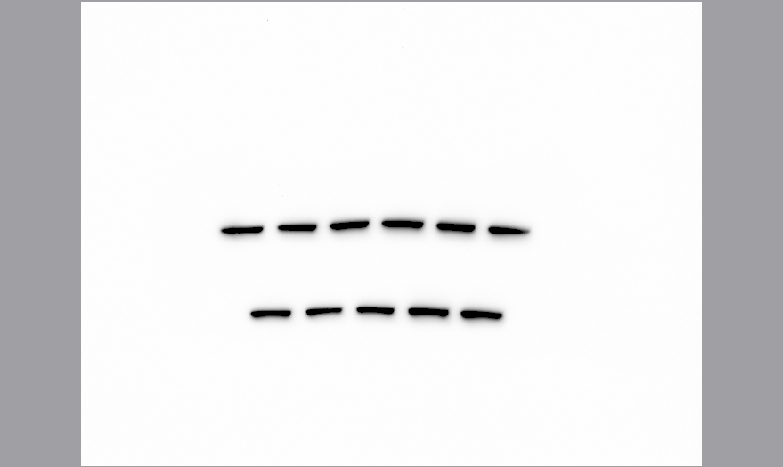

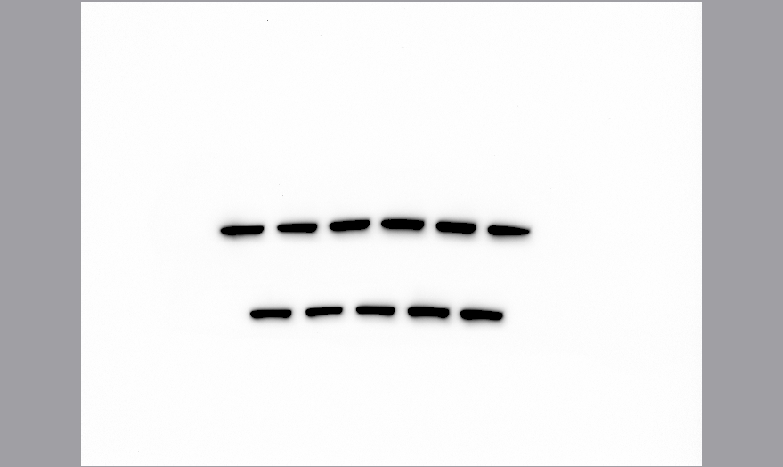

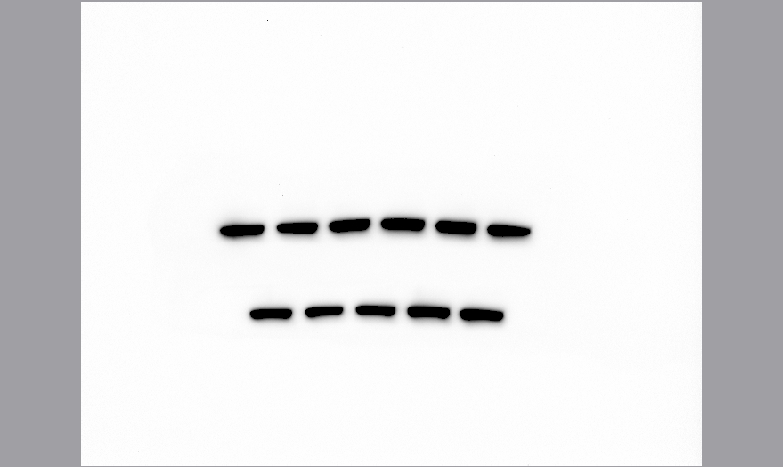


Fig 6A

β-actin

Fig 6A

β-actin

Fig 6A

β-actin

Fig 6A

β-actin

1. The bands above were the multiple exposure images of β-actin in Figure 6A of the text.


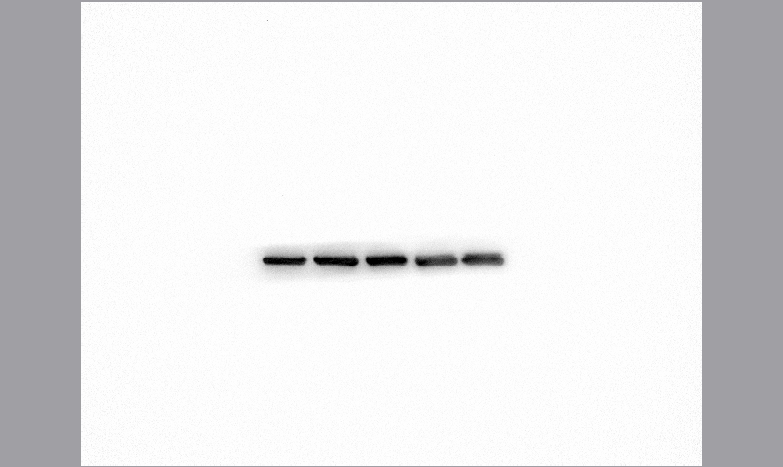

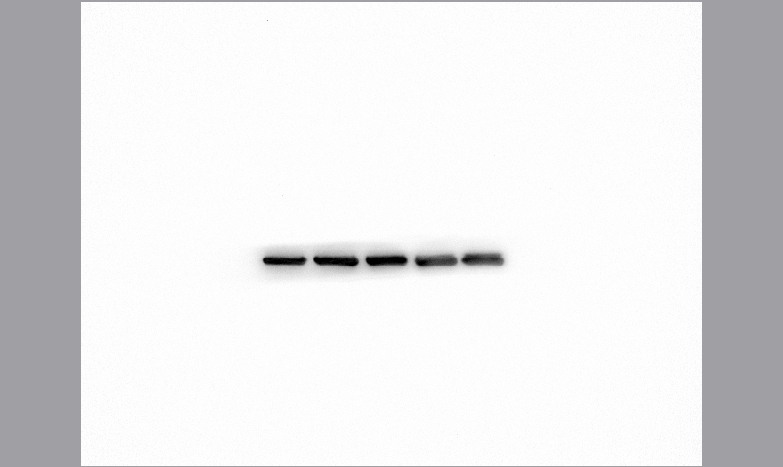

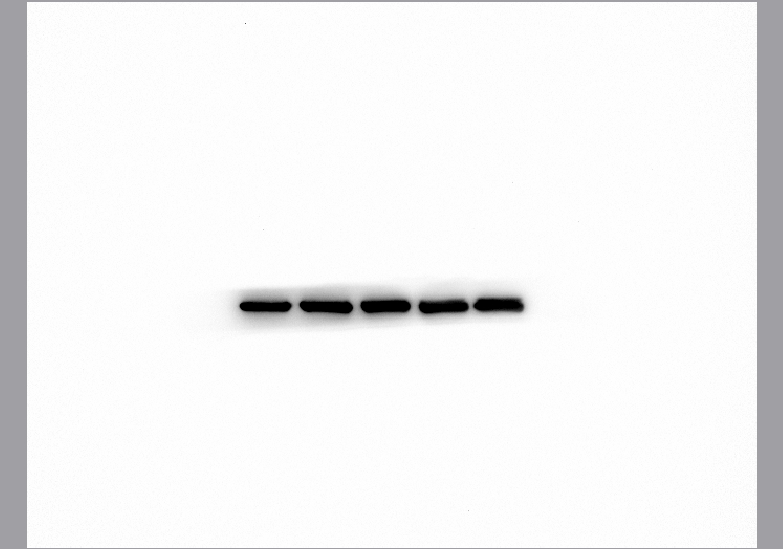


Fig 6E

β-actin

Fig 6E

β-actin

Fig 6E

β-actin

1. The multiple exposure images of β-actin in Figure 6E of the text.
