# Supplementary material for: Notch1 activation of Jagged1 contributes to differentiation of mesenchymal stem cells into endothelial cells under cigarette smoke extract exposure
Source: BMC Pulm Med. 2022 Apr 11;22:139. doi: 10.1186/s12890-022-01913-3 (PMC9004089; doi:10.1186/s12890-022-01913-3)
Supplement: Supplementary file 2 — Additional file 2. MSCs characterization. [file 12890_2022_1913_MOESM2_ESM.docx]

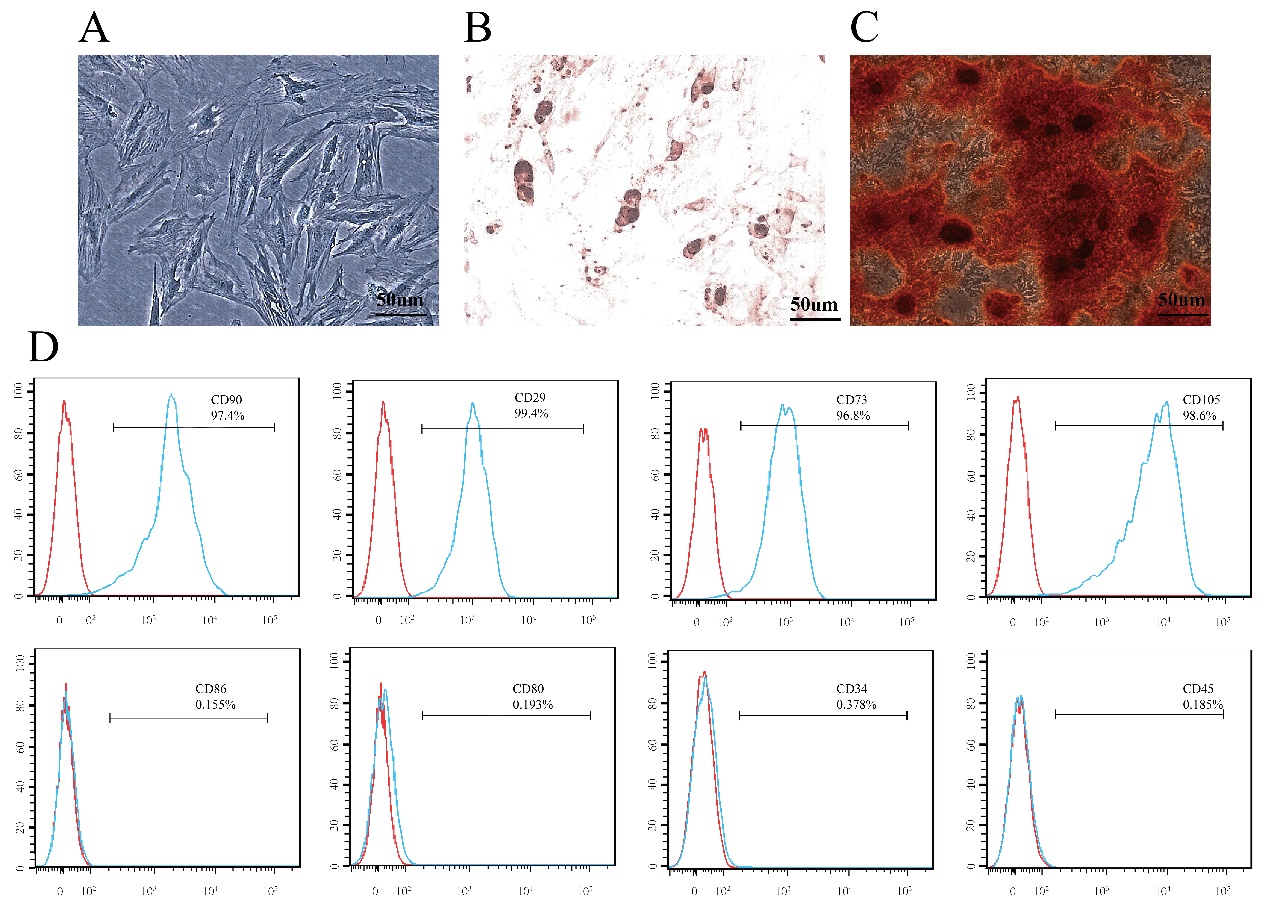


Fig. S1. Isolated mesenchymal stem cells (MSCs) exhibited a fibroblast-like shape (A), exhibited adipocyte (B) and osteoblast (C) differentiation capacity. MSCs were phenotypically characterized by flow cytometry. The results demonstrated that MSCs were uniformly negative for CD86, CD80, CD34, and CD45, and positive for CD90, CD29, CD73, and CD105 expression (D).
