# Supplementary material for: Notch1 activation of Jagged1 contributes to differentiation of mesenchymal stem cells into endothelial cells under cigarette smoke extract exposure
Source: BMC Pulm Med. 2022 Apr 11;22:139. doi: 10.1186/s12890-022-01913-3 (PMC9004089; doi:10.1186/s12890-022-01913-3)
Supplement: Supplementary file 3 — Additional file 3. The original western blot images of β-actin. [file 12890_2022_1913_MOESM3_ESM.docx]

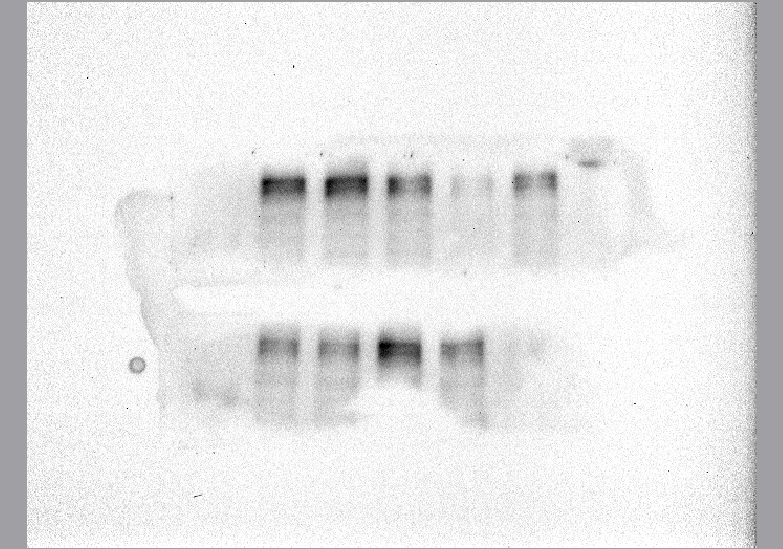


Fig2 A

N1ICD


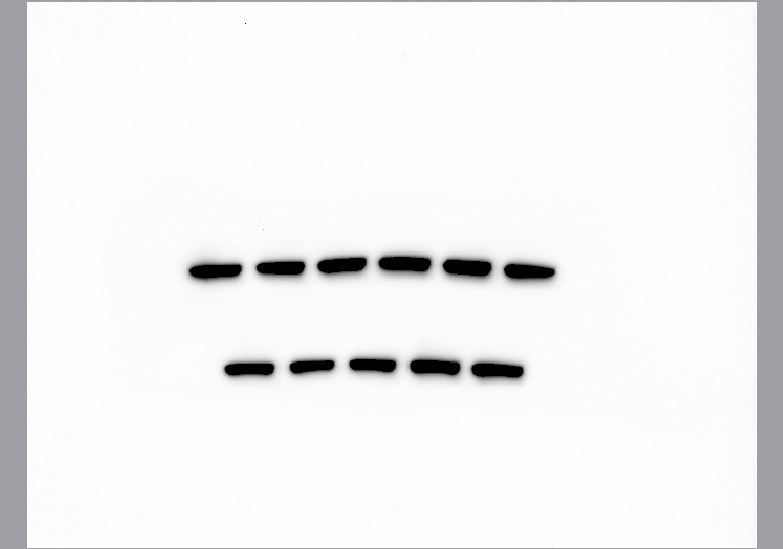

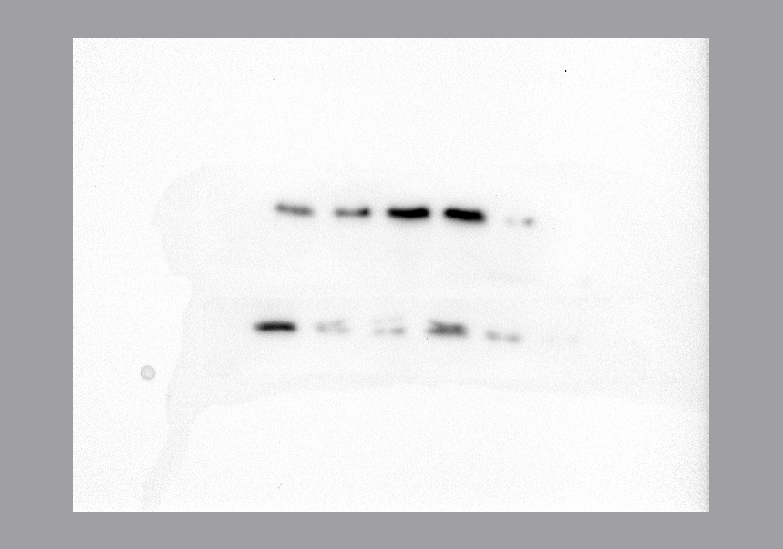

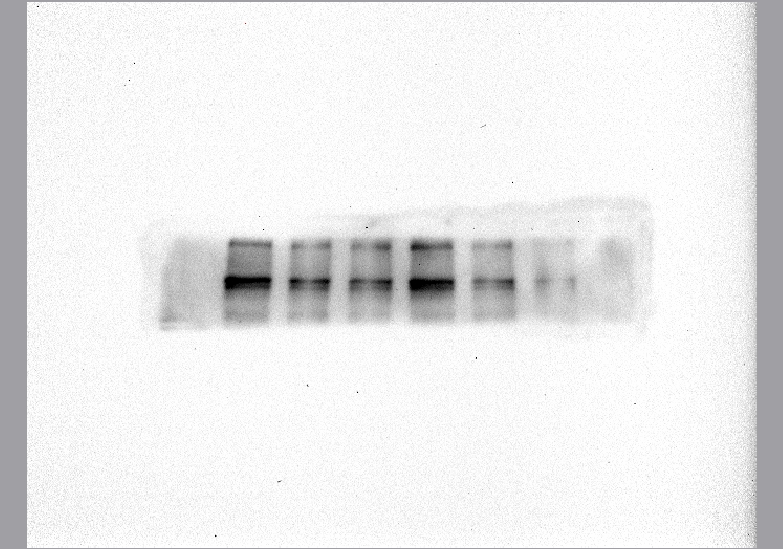

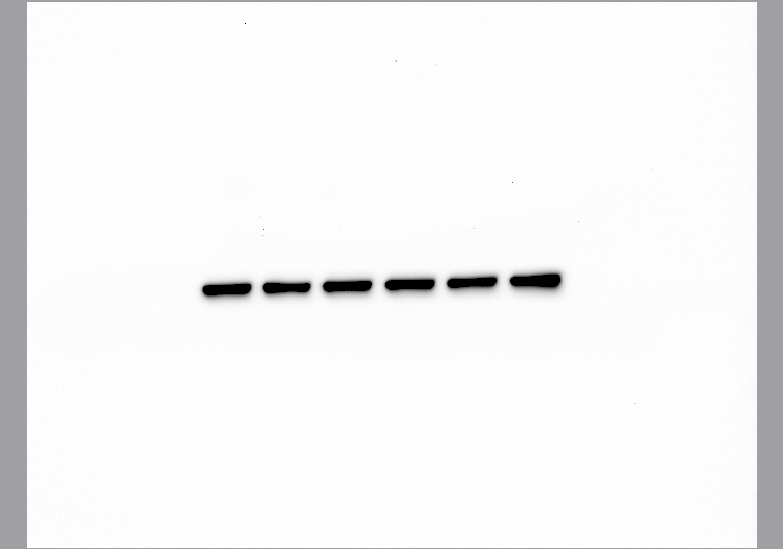

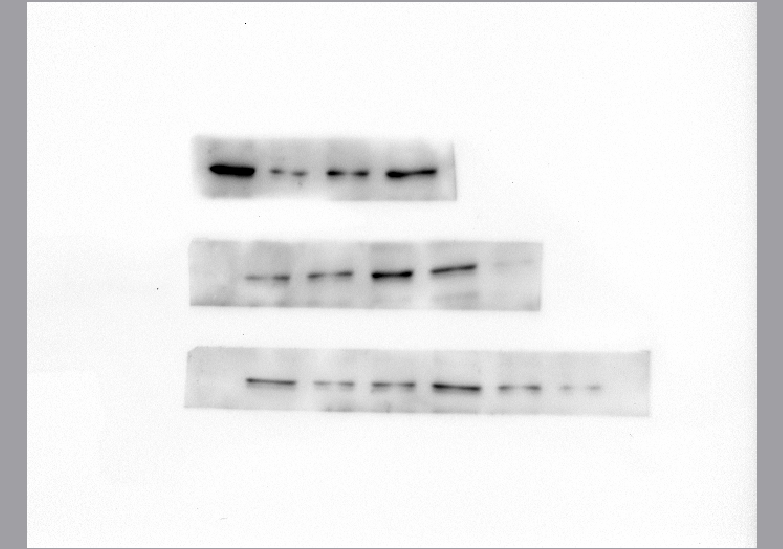

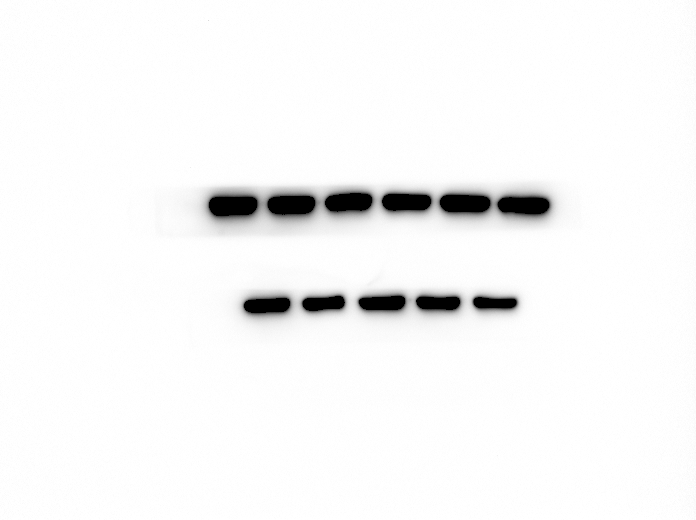


Fig2 A

Β-actin

Fig2 G

Hey-1

Fig2 G

JAG-1

Fig2 G

Β-actin

Fig3 C

VEGFR2

Fig3 C

β-actin


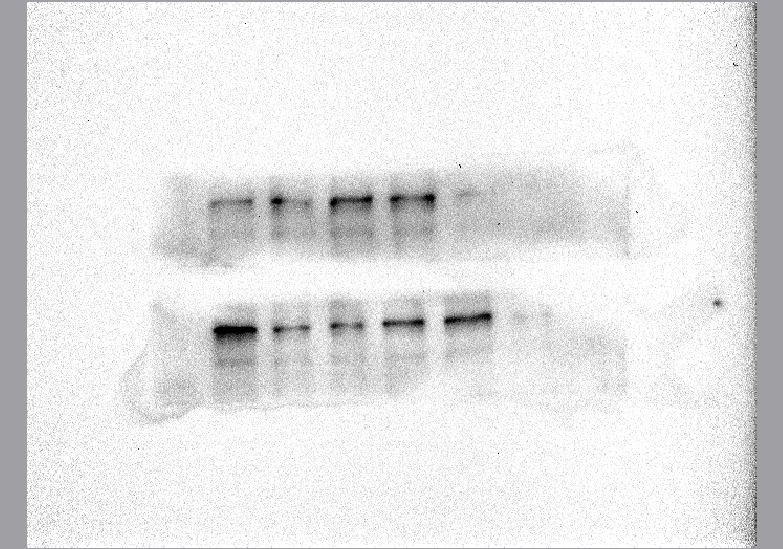

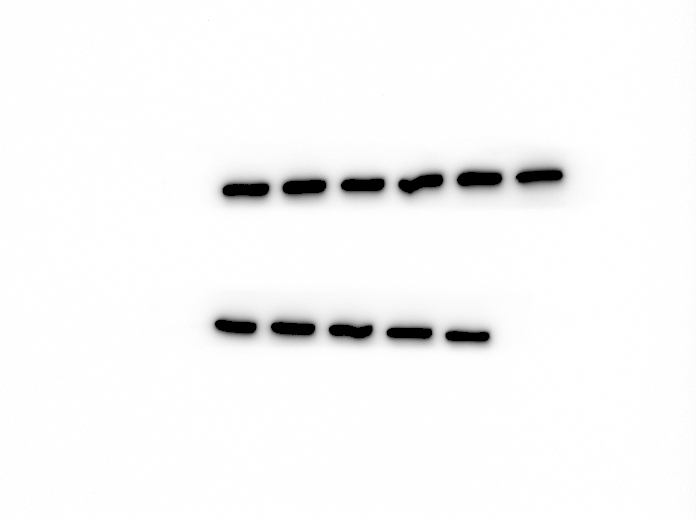

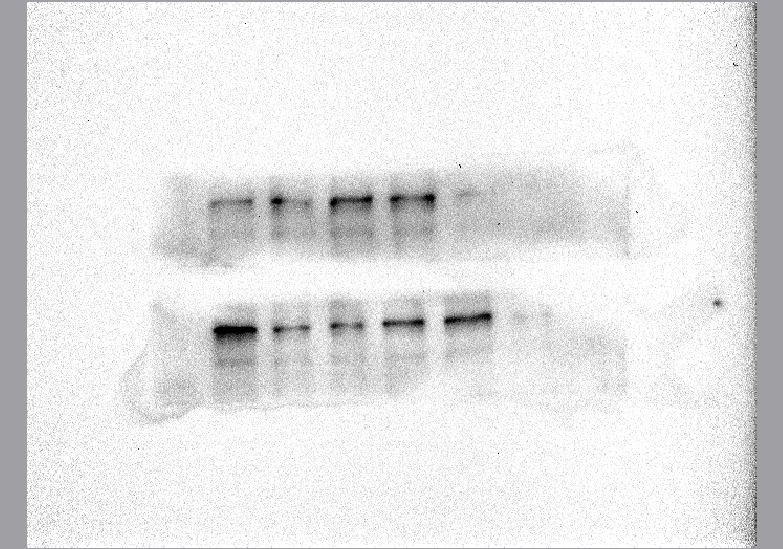


Fig4 B

β-actin

Fig4 B

JAG1

Fig4 F

JAG1


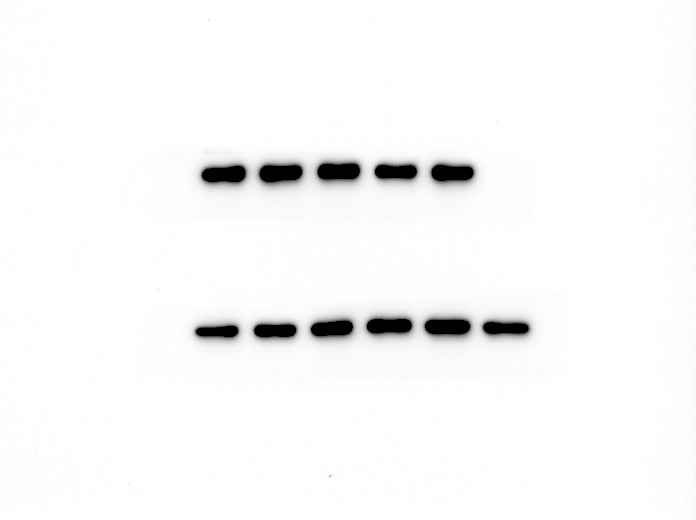


Fig4 F

β-actin


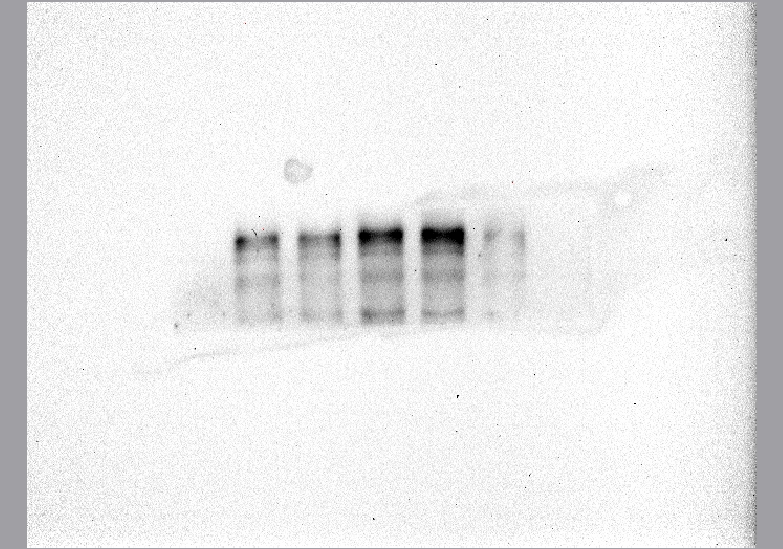


Fig5A

N1ICD


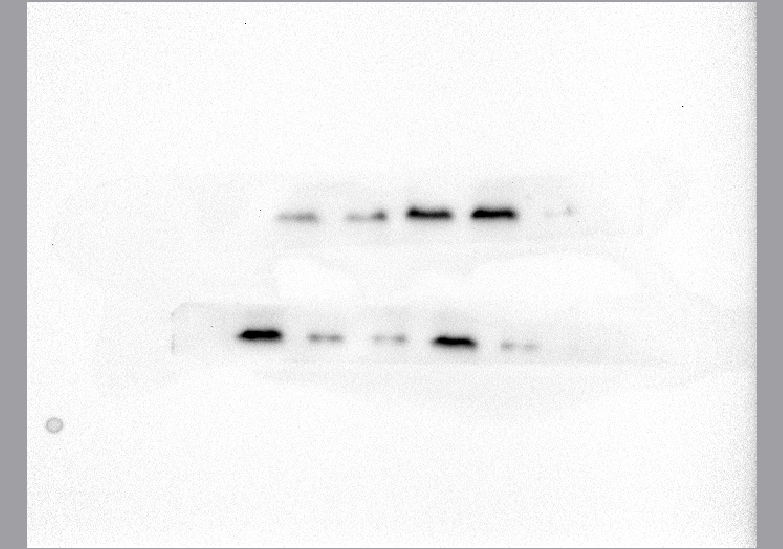


Fig5A

Hey 1


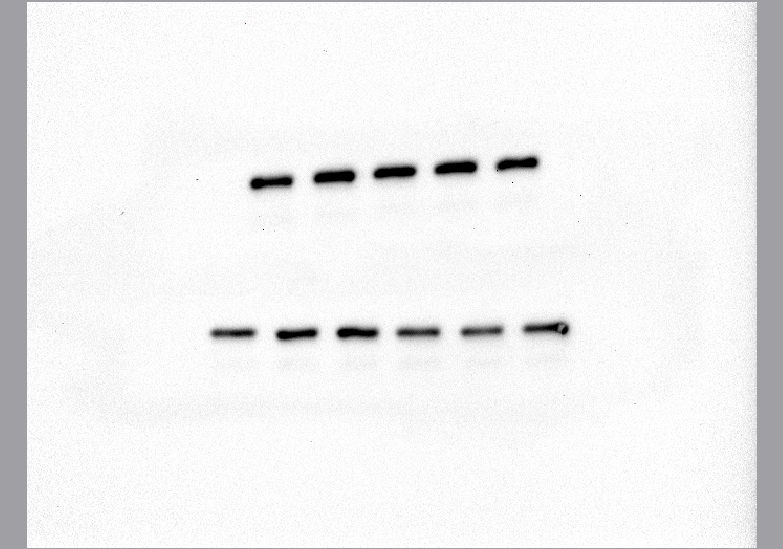


Fig5A

β-actin


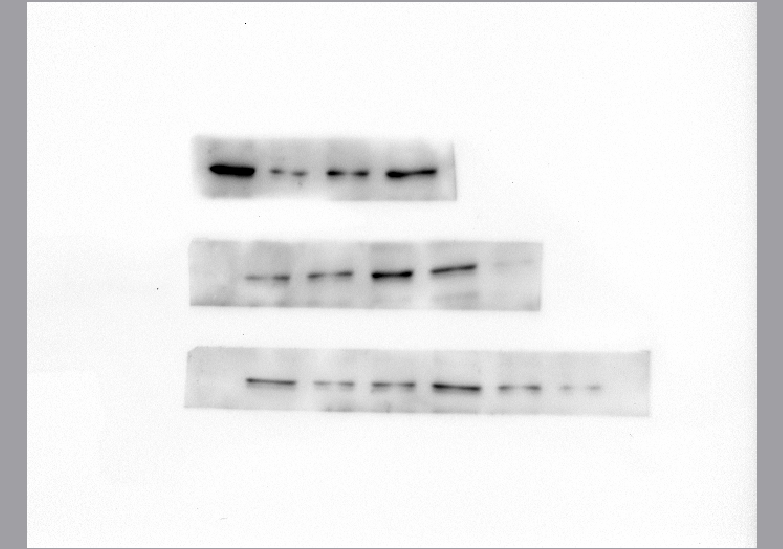


Fig5C

VEGFR2


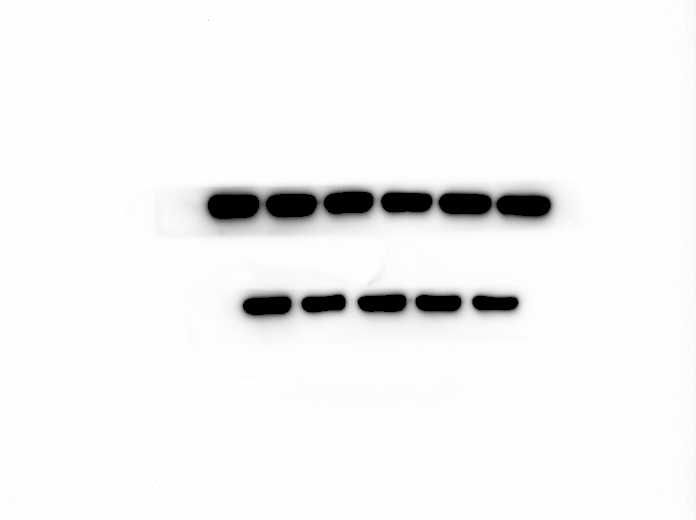


Fig5C

β-actin


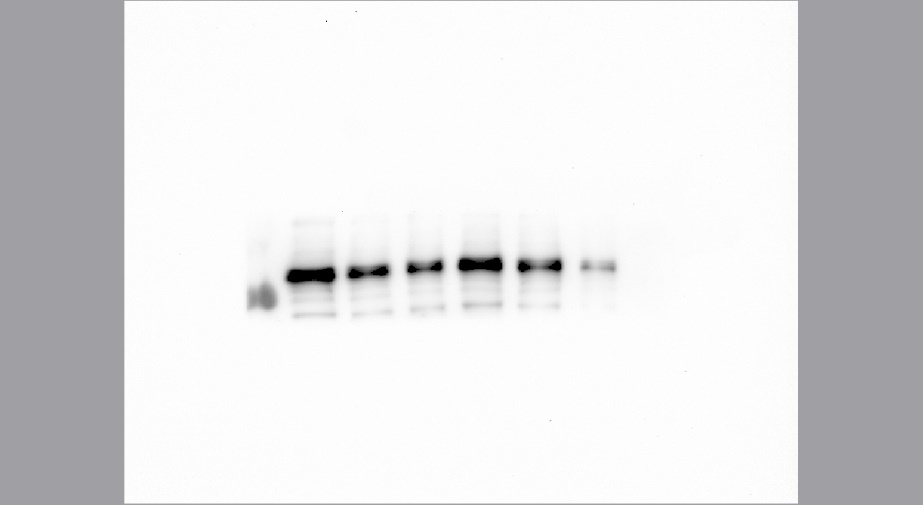


Fig6A

Hif 1α


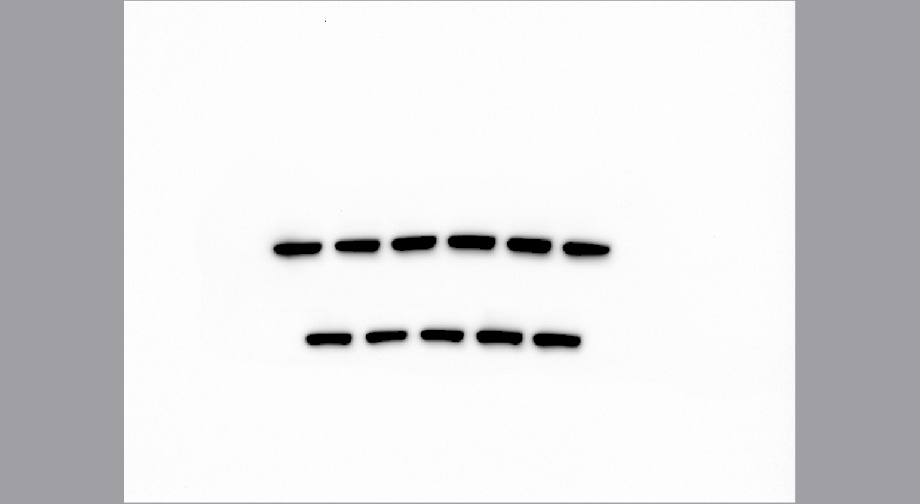


Fig6A

β-actin


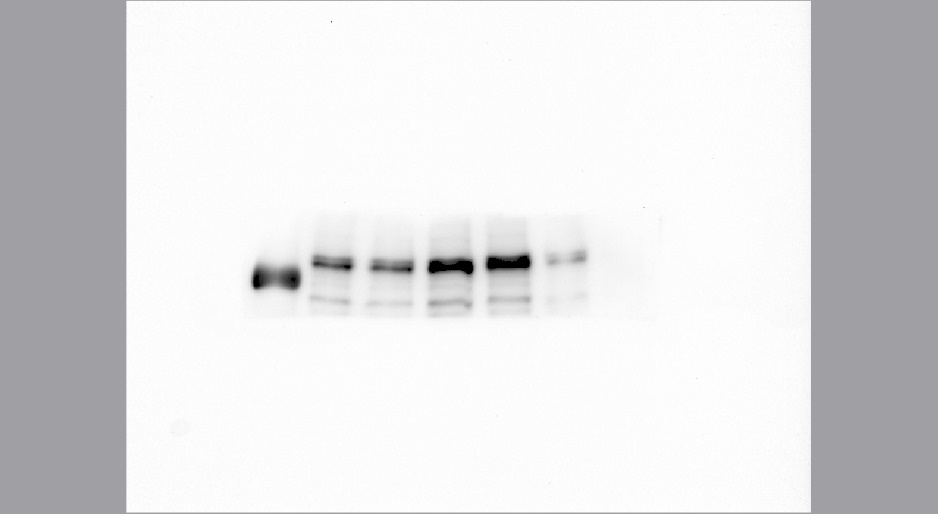


Fig6E

Hif 1α


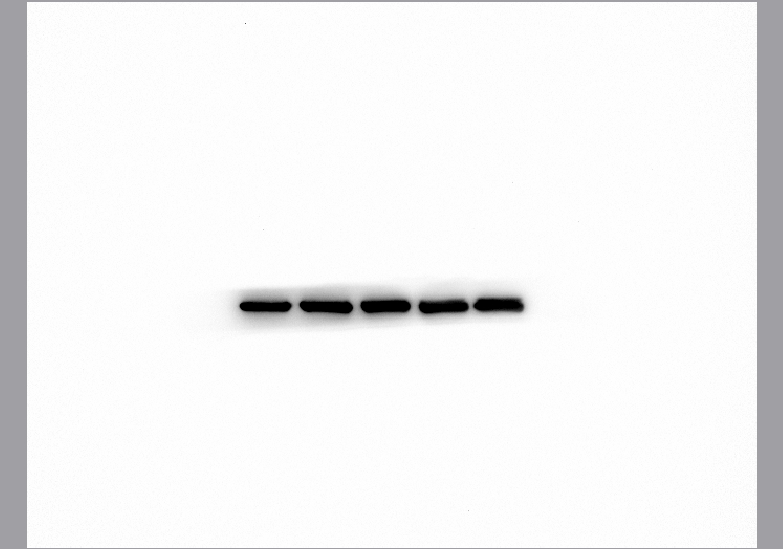


Fig6E

β-actin
